# Ocular Expression and Distribution of Products of the POAG-Associated Chromosome 9p21 Gene Region

**DOI:** 10.1371/journal.pone.0075067

**Published:** 2013-09-19

**Authors:** Glyn Chidlow, John P. M. Wood, Shiwani Sharma, David P. Dimasi, Kathryn P. Burdon, Robert J. Casson, Jamie E. Craig

**Affiliations:** 1 Ophthalmic Research Laboratories, South Australian Institute of Ophthalmology, Hanson Institute Centre for Neurological Diseases, Adelaide, South Australia, Australia; 2 Department of Ophthalmology and Visual Sciences, University of Adelaide, Adelaide, South Australia, Australia; 3 Department of Ophthalmology, Flinders University, Bedford Park, South Australia, Australia; Sanjay Gandhi Medical Institute, India

## Abstract

It has recently been shown that there are highly significant associations for common single nucleotide polymorphisms (SNPs) near the *CDKN2B-AS1* gene region at the 9p21 locus with primary open angle glaucoma (POAG), a leading cause of irreversible blindness. This gene region houses the *CDKN2B/p15^INK4B^*_,_ *CDKN2A/p16^INK4A^* and *p14ARF* (rat equivalent, *p19^ARF^*) tumour suppressor genes and is adjacent to the S-methyl-5′-thioadenosine phosphorylase (*MTAP*) gene. In order to understand the ocular function of these genes and, therefore, how they may be involved in the pathogenesis of POAG, we studied the distribution patterns of each of their products within human and rat ocular tissues. *MTAP* mRNA was detected in the rat retina and optic nerve and its protein product was localised to the corneal epithelium, trabecular meshwork and retinal glial cells in both human and rat eyes. There was a very low level of *p16^INK4A^* mRNA present within the rat retina and slightly more in the optic nerve, although no protein product could be detected in either rat or human eyes with any of the antibodies tested. *P19^ARF^* mRNA was likewise only present at very low levels in rat retina and slightly higher levels in the optic nerve. However, no unambiguous evidence was found to indicate expression of specific P19^ARF^/p14^ARF^ proteins in either rat or human eyes, respectively. In contrast, *p15^INK4B^* mRNA was detected in much higher amounts in both retina and optic nerve compared with the other genes under analysis. Moreover, p15^INK4B^ protein was clearly localised to the retinal inner nuclear and ganglion cell layers and the corneal epithelium and trabecular meshwork in rat and human eyes. The presented data provide the basis for future studies that can explore the roles that these gene products may play in the pathogenesis of glaucoma and other models of optic nerve damage.

## Introduction

Glaucoma is a leading cause of irreversible blindness and manifests as an age-related, progressive optic neuropathy with a poorly understood pathogenesis and limited treatment options [1]. It affects approximately 2.5% of the general population over 40 years of age, with the prevalence increasing exponentially thereafter [2], [3]. The most common form of glaucoma is primary open-angle glaucoma (POAG), which has a poorly understood cause and pathogenesis but which makes up approximately 74% of all cases [3]. This particular form of glaucoma is usually treated by lowering intraocular pressure (IOP). Although such a treatment regimen can delay progression of the disease in many cases, patients often present only after irreversible retinal damage has occurred. Therefore, further research is essential to address the underlying causes of POAG.

In recent years, researchers have attempted to elucidate pathological mechanisms involved in the etiology of POAG by identifying familial links that may indicate a genetic basis for this disease. The first gene to be identified as such was *MYOC* which encodes the myocilin protein in trabecular meshwork (TM) cells [4], [5]. Indeed, *MYOC* mutations are now known to underlie 3–5% of POAG cases. Other genes identified in a similar manner to play potential roles in glaucoma pathogenesis are the *OPTN* [6], the *NTF4* [7] and the *WDR36* [4] genes.

A related analytical tool employed to aid elucidation of the pathogenesis of POAG has been the genome-wide association study (GWAS). This method determines the prevalence of single nucleotide polymorphisms (SNPs) at distinct genetic loci which are significantly associated with a particular disease: in this case, POAG. The first SNP location reproducibly identified using a GWAS approach was the *CAV1*/*CAV2* locus on chromosome 7q34, which encodes the caveolin 1 and caveolin 2 proteins [5], [6]. More recent studies identified significant genome-wide association with POAG at the rs4656461 SNP near the *TMCO1* gene (encoding transmembrane and coiled-coil domain-containing protein 1; TMCO1) on chromosome 1q24 [7], [8], [9]. Subsequent analysis of TMCO1 expression within the human eye showed that although having an unknown function, this protein localised to nuclear inclusions (nucleoli) in most tissue regions, including both the retina and trabecular meshwork, implying a role in cellular control [10].

**Figure 1:**
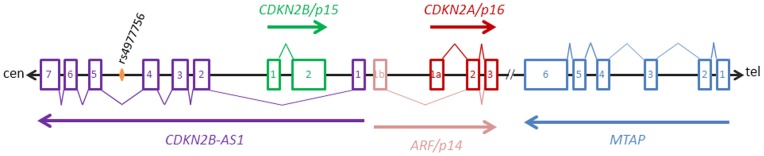
Genomic organisation of the human 9p21 gene cluster. Organisation of the *CDKN2A/ARF*, *CDKN2B* and *CDKN2B-AS1* genes at the 9p21 locus and the neighbouring gene, *MTAP*. The boxes represent exons, numbers in boxes correspond to exon numbers, lines between boxes represent introns/intergenic regions, V-lines represent exon-splicing, arrows represent encoded transcript/protein and double-slash indicates large intergenic region. The two gene products, CDKN2A and ARF, encoded by the *CDKN2A/ARF* gene are respectively represented in red and light red. The SNP most significantly associated with open-angle glaucoma in the *CDKN2B-AS1* gene is represented by an orange diamond. Note: the figure is not drawn to scale.

The most significant region to be identified, by independent research groups, as having an association with POAG in different population samples is the CDKN2B-AS1 region on chromosome 9p21 [7], [8], [11], [12], [13], [14], [15], [16], [17], [18], [19], [20]. The significance of this finding derives from the fact that SNPs which affect genetic expression in the region of *CDKN2B-AS1* are known to be associated with a number of diseases such as coronary heart disease [21], [22], [23], types 1 and 2 diabetes [21], [24], [25], atherosclerosis [26] and different cancers (see reviews by Pasmant *et al*., 2011 and Cunnington *et al*., 2010 [24], [25]). *CDKN2B-AS1* or ANRIL (anti-sense non-coding RNA at the INK4 locus) encodes an extended non-coding RNA spanning 19 exons which resides in the CDKN2B/p15^INK4B^-CDKN2A/p16^INK4A^-p14^ARF^ tumor suppressor gene cluster [27] (see figure 1). This RNA gene is also adjacent to the *MTAP* gene, which encodes the protein S-methyl-5′-thioadenosine phosphorylase (MTAP) [28]. The proteins p15^INK4B^ and p16^INK4A^ are involved in cellular proliferation and senescence by inhibiting cyclin-D binding at cell cycle-regulating cyclin-dependent kinases [29]. Human p14^ARF^ (rat homologue is p19^ARF^) is transcribed from an alternate reading frame of the p16^INK4A^ locus in response to sustained mitogenic stimulation, and is involved with nucleolar regulation of ribosome biosynthesis, initiation of p53-dependent cell cycle arrest and apoptosis [36], [37]. MTAP is involved with polyamine, methionine and adenine metabolism [28]. Thus, SNPs causing altered expression of any of the genes derived from this region of the 9p chromosome may have serious pathological consequences.

Since it is known that SNPs associated with the *CDKN2B-AS1* gene region effectively comprise a major “hotspot” that relates to many diseases [24], [25], then the finding that this region is strongly and reproducibly associated with POAG is extremely significant. The present study therefore sought to investigate the ocular expression and localisation patterns of products from the *CDKN2B-AS1* gene region. Detailed information on the previously unstudied ocular expression profiles of the gene products encoded by these loci, is likely to be of great importance in determining their role in glaucoma pathogenesis.

## Materials and Methods

### Tissue Collection

Adult Sprague-Dawley (SD) rats (8–10 weeks old) were obtained from The University of Adelaide and were housed according to the Australian Code of Practice for the Care and Use of Animals for Scientific Purposes, 2004, and the Association for Research in Vision and Ophthalmology (ARVO) Statement for the Use of Animals in Ophthalmic and Vision Research. The work was approved by the South Australia (SA) Pathology/Central Northern Health (CNH) Animal Ethics Committee. No actual experimental procedures were carried out on animals: when tissue was required, all tissues were procured by rapid killing of animals under terminal anaesthesia by trans-cardiac perfusion with 0.9% (w/v) NaCl solution to achieve exsanguination. In some cases, rat liver and brain cortex samples were also prepared (for Western immunoblot or reverse-transcription polymerisation chain reaction; RT-PCR). For some optic nerve analysis, pigmented inbred Dark Agouti (DA) rats (8–10 weeks old) were also analysed: samples from these animals were prepared in the same way as for the standard SD rats. For each antibody tested, and each analytical method used, at least 6 animals were assessed. Human ocular tissue for analysis was obtained from the Eye-Bank of South Australia, Flinders Medical Centre (Adelaide, Australia) following the guidelines of the Southern Adelaide Clinical Human Research Ethics Committee; all had been screened to make sure there was no underlying ocular disease and all were from Caucasian donors between the ages of 50 and 65. Tissue sections of malignant pleural mesothelioma and cervical adenocarcinoma, which served as positive controls in the present study, were sourced from positive control blocks provided by the Surgical Diagnostic facility of SA Pathology (Adelaide, Australia).

### Antibodies

A review of the relevant literature revealed that each of the protein products to be analysed (MTAP, p14^ARF^ [human tissue], p19^ARF^ [rat tissue], p16^INK4A^, p15^INK4B^) have had their expression in various tissues determined by the use of a number of antibodies. It was therefore decided in the interests of accuracy and consistency to use a selection of the most frequently used antibodies for each protein under analysis, in order to determine the most reproducible expression/localisation data. Antibody data is summarised in Table 1.

**Table 1:** Antibodies used for detection of 9p21-related gene products.

Protein Species	Antibody	Clone/Cat. No.	Species	Concentration	Dilution
**MTAP**	Proteintech	1475-1-AP	Rabbit	133 µg/ml	1∶800, 1∶2000^W^
	Santa Cruz	sc-100782	Mouse	100 µg/ml	1∶100, 1∶1000^W^
	CST	#4158	Rabbit	13.5 µg/ml	1∶100, 1∶1000^W^
**p14^ARF^**	Santa Cruz	sc-8340	Rabbit	200 µg/ml	1∶1000, 1∶1000^W^
	Sigma	DCS-240	Mouse	2 mg/ml	1∶1000, 1∶2000^W^
**p19^ARF^**	Upstate	07-543	Rabbit	100 µg/ml	1∶100, 1∶1000^W^
	Santa Cruz	G-19	Goat	200 µg/ml	1∶100, 1∶2000^W^
**p15^INK4B^**	Santa Cruz	K-18	Rabbit	200 µg/ml	1∶500, 1∶500^W^
	Neomarkers	†15P06	Mouse	200 µg/ml	1∶500, 1∶1000^W^
	CST	#4822	Rabbit	34 µg/ml	1∶1000, 1∶1000^W^
	Abcam	Ab53034	Rabbit	1 mg/ml	1∶1000, 1∶1000^W^
**p16^INK4A^**	Santa Cruz	‡sc-1661	Mouse	n/a	n/a
	Proteintech	10883-1-AP	Rabbit	146 µg/ml	1∶500, 1∶1000^W^
	Sigma	4C11	Mouse	1 mg/ml	1∶500, 1∶1000^W^
	Abnova	2D9A12	Mouse	1 µg/ml	1∶2000, 1∶1000^W^

W dilution used for Western blotting; CST, Cell Signalling Technology^;^

† discontinued product;

‡ sc-1661 is exclusively under licence to CINtec® within Australia as the primary antibody component of an immunohistochemistry assay for detection of p16^INK4A^ in cervical tissue sections.

Other un-related antibodies (eg. β-actin, Ret-P1 for rhodopsin, α-tubulin, FGF-2, vimentin, CD45, glutamine synthetase) were used as described previously [30], with appropriate dilutions recorded where employed.

### Recombinant Proteins as Positive Controls

To test the specificity for each antibody under investigation, recombinant proteins for each of the species under test were obtained. These were to enable positive reactivity testing and pre-adsorption neutralisation for each relevant antibody. A human-specific protein/peptide for p14^ARF^ was unable to be obtained, although a rodent-specific version (p19) was procured. p19^ARF^ peptide was from Abcam (Cambridge, UK; #ab2659; 5 mg/ml) and p16^INK4A^, p15^INK4B^ and MTAP proteins were obtained from Abnova (Walnut, CA, USA). The latter three products were obtained as GST-fusion products; hence these peptides had molecular masses that were 26 kD larger than reported values in each case (due to the additional mass of GST). Details: p15^INK4B^, human sequence with full length ORF (residues 1–138), #H00001030-P01, 0.04 µg/ml; p16^INK4A^, human sequence with full length ORF (residues 1–105), #H00001029-P01, 0.25 µg/ml; MTAP, human sequence with full length ORF (residues 1–283), #H00004507-P01, 0.09 µg/ml.

### Tissue Processing and Immunohistochemistry

Whole eyes plus attached optic nerves that were subsequently used for immunohistochemistry were immersion-fixed in either 10% (w/v) neutral buffered formalin for 24–72 h until processing, or in Davidson’s solution (2 parts 37% formaldehyde, 3 parts 100% ethanol, 1 part glacial acetic acid and 3 parts water) for 24 h followed by 70% ethanol until processing. Davidson’s solution is the preferred fixative for whole eyes as it provides optimal tissue preservation without artefactual retinal detachment. Eyes were subsequently hand-processed according to the following schedule: 70% ethanol (30 min), 3×100% ethanol (30 min), 2×xylene (30 min), 50% xylene/50% wax (30 min) at 62°C, 2×wax (30 min) at 62°C. Globes were embedded in a sagital orientation and 4 µm sections were cut using a rotary microtome.

Immunohistochemistry of transverse ocular sections was performed as previously described [30], [31]. Briefly, tissue sections were deparaffinised and endogenous peroxidase activity was blocked with H_2_O_2_. Antigen retrieval was performed by microwaving sections in 1 mM EDTA (pH 8.0) and non-specific labelling blocked with PBS containing 3% normal horse serum (PBS-HS). Sections were incubated overnight at room temperature in primary antibody (in PBS-HS), followed by consecutive incubations with biotinylated secondary antibody (Vector, Burlingame, CA) and streptavidin-peroxidase conjugate (Pierce, Rockford, IL). Color development was achieved using NovaRed substrate kit (Vector, Burlingame, CA) for 3 min. Sections were counterstained with hematoxylin, dehydrated, cleared in histolene and mounted in DPX. In order to evaluate specificity of antibody labelling, adjacent sections were incubated with the appropriate isotype control for monoclonal antibodies, or normal rabbit/goat serum for polyclonal rabbit/goat antibodies. In addition, Western blotting was performed for the majority of the antibodies used.

For antibody-antigen neutralisation studies, antibodies were pre-adsorbed with or without a ten-fold excess of the appropriate recombinant protein in PBS-HS for three hours on a rotary shaker at room temperature before being applied to sections in a standard manner.

### Western Immunoblotting

Dissected tissue was sonicated in homogenisation buffer (20 mM Tris-HCl, pH 7.4, 25°C; with 2 mM EDTA, 0.5 mM EGTA, 1 mM dithiothreitol, 50 µg/ml leupeptin, 50 µg/ml pepstatin A, 50 µg/ml aprotinin and 0.1 mM phenylmethylsulphonyl fluoride). An equal volume of sample buffer (62.5 mM Tris-HCl, pH 7.4, containing 4% SDS, 10% glycerol, 10% β-mercaptoethanol and 0.002% bromophenol blue) was subsequently added and samples were boiled for 5 minutes. In order to ensure even running of samples on gels, protein concentrations for each tissue extract were equalized according to the method of Bradford [32].

Electrophoresis was performed using a Mini-PROTEAN® TGX™ precast gel system (Bio-Rad Laboratories Pty Ltd, Gladesville, New South Wales, Australia). Denaturing 12% (w/v) polyacrylamide gels were employed for protein separation, as described previously [33]. Separated proteins were transferred to polyvinylidine fluoride membranes (PVDF; Bio-Rad Laboratories Pty Ltd) for labelling. Membranes were incubated with appropriate primary antibody overnight, and actual labelling of immunoblots carried out using a two-step detection procedure: first, appropriate biotinylated secondary antibodies were applied (Vector Laboratories; 1∶500; 30 minutes) and subsequently streptavidin-peroxidase conjugates (Pierce, Rockford, Il, USA). Blocking of membranes was carried out in a solution of tris-buffered saline (TBS) containing 0.1% (v/v) Tween-20 and 5% (w/v) non-fat dried skimmed milk (TBS-TM). Positive antibody labelling was detected as described previously [39] and quantification of detected proteins was achieved, where appropriate, using the program, Adobe PhotoShop CS2. Detection of β-actin was assessed where suitable as a positive gel-loading control.

For Western immunoblot testing of specificity of each antibody to recognise the appropriate antigen, recombinant proteins were resolved on 12% SDS-PAGE gels after loading at either 100 ng or 10 ng per lane before being detected as described above.

### Real-time PCR

Real time PCR studies were carried out essentially as described previously [30], [31]. In brief, the required tissues were dissected, total RNA was isolated and first strand cDNA was synthesised from DNase-treated RNA samples. Real-time PCR reactions were carried out in 96-well optical reaction plates using the cDNA equivalent of 20 ng total RNA for each sample in a total volume of 25 µl containing 1×SYBR Green PCR master mix (BioRad), forward and reverse primers. Thermal cycling conditions were 95°C for 3 min and 40 cycles of amplification comprising 95°C for 12 s, appropriate annealing temperature for 30 s and 72°C for 30 s. Primers employed for the study are outlined in Table 2. To allow a comparison to be made between the levels of expression of test mRNAs in the different tissues, results were semi-quantified using the Relative Expression Software Tool (REST©). Threshold cycles were calculated using IQ5 icycler Software (Bio-Rad), all values were normalised using the endogenous reference gene, cyclophilin, and results expressed as mean ± SEM.

**Table 2:** Primer Sequences for mRNAs Amplified by Real-Time RT-PCR.

mRNA	Primer sequences	Productsize (bp)	Mg^2+^conc^n^	Annealingtemperature	Accessionnumber
cyclophilin	5′-GTGTTCTTCGACATCACGGCT-3′	82	3 mM	63°C	NM_017101
	5′-CTGTCTTTGGAACTTTGTCTGCA-3′				
MTAP (set 1)	5′-TGGAATAATTGGTGGAACAGGC-3′	513	3.5 mM	62°C	NM_001047867
	5′-TGGCACACTCCTCTGGCAC-3′				
MTAP (set 2)	5′-AGTGTCAGTGGATGGGGTTT-3′	122	3.5 mM	62°C	NM_001047867
	5′-TTAGGTTACGGAGCGTTTCTG-3′				
p15^INK4B^	5′-AGATCCCAACGCCGTCAAC-3′	184	3.5 mM	61°C	NM_130812
	5′-CAGCACCATTAGCGTGTCCAG-3′				
p16^INK4A^/p19^ARF^	5′-CGTGCGGTATTTGCGGTATCT-3′	171	3.5 mM	61°C	NM_031550
	5′-GCCAGAAGTGAAGCCAAGGA-3′				
p16^INK4A^	5′-TGCAGATAGACTAGCCAGGGC-3′	185	3.5 mM	59°C	NM_031550
	5′-CTCGCAGTTCGAATCTGCAC-3′				
p19^ARF^	5′-GGTCGCAGGTTCGTGGTC-3′	412	3.5 mM	61°C	AY679727
	5′-GTCGTGATGTCCCCGCTCT-3′				

### RGC-5 Cell-Line

This murine cell-line [34] comprises retina-derived cells which can be differentiated into neurons [35]. They were cultivated exactly as described previously [36]. Their use in the present study was to provide a positive control preparation for identification of P19^ARF^.

### Immunocytochemical Analysis of Cultures

Cells on coverslips were fixed with neutral-buffered formalin containing 1% methanol for 15 minutes and then washed in phosphate buffered saline (PBS; 137 mM NaCl, 5.4 mM KCl, 1.28 mM NaH_2_PO_4_, 7 mM Na_2_HPO_4_; pH 7.4). Cells were permeabilised with PBS containing 0.1% (v/v) Triton X-100 (PBS-T), followed by further washing in PBS and then blocking in PBS-HS. Test antisera (see Table 1), diluted in PBS-HS, were applied overnight at room temperature and labelling completed with consecutive incubations in appropriate biotinylated secondary antibodies (Vector Laboratories, Abacus ALS, Brisbane, Australia; 1∶250 in PBS-HS; 30 minutes) and streptavidin-AlexaFluor 488 or streptavidin-AlexaFluor 594 (Molecular Probes, Invitrogen, Mulgrave, Victoria, Australia; 1∶500 in PBS-HS; 1 hour). Cells on coverslips were mounted using anti-fade mounting medium (DAKO, Botany, New South Wales, Australia) and examined under a confocal fluorescence microscope.

## Results

### MTAP

#### RT-PCR

Table 3 shows the expression level of MTAP mRNA, relative to the housekeeping gene cyclophilin, in four rat tissues (brain cortex, liver, optic nerve and retina) using two distinct sets of primers to ensure reproducibility. The first point to note is that there was a good agreement between the two primer sets. Regarding abundance, there was a broadly similar level of MTAP mRNA across the different tissues. The MTAP transcript is present at approximately 0.3% and 1% of the level of glyceraldehyde-3-phosphate dehydrogenase and cyclophilin, respectively, in retina and optic nerve, with somewhat higher and lower levels present in the liver and brain samples, respectively.

**Table 3:** Expression of MTAP mRNA in rat tissues.

		retina	optic nerve	brain	liver
cyclophilin	C_T_	17.6±0.1	19.5±0.2	18.2±0.1	19.5±0.2
MTAP	C_T_	24.6±0.1	27.0±0.2	26.6±0.1	26.5±0.3
(primer set 1)	1ΔC_T_	7.0±0.1	7.0±0.1	8.4±0.1	7.1±0.0
	2mRNA level	0.010±0.001	0.010±0.001	0.004±0.000	0.009±0.002
MTAP	C_T_	24.6±0.1	26.7±0.1	26.2±0.1	25.8±0.3
(primer set 2)	1ΔCT	6.9±0.1	6.7±0.1	8.0±0.0	6.3±0.0
	2mRNA level	0.010±0.001	0.012±0.002	0.005±0.000	0.015±0.003

C_T_, cycle threshold;

1 ΔCT, target C_T_ - cyclophilin C_T_;

2 target mRNA level expressed relative to cyclophilin.

#### Antibody validation

**Figure 2:**
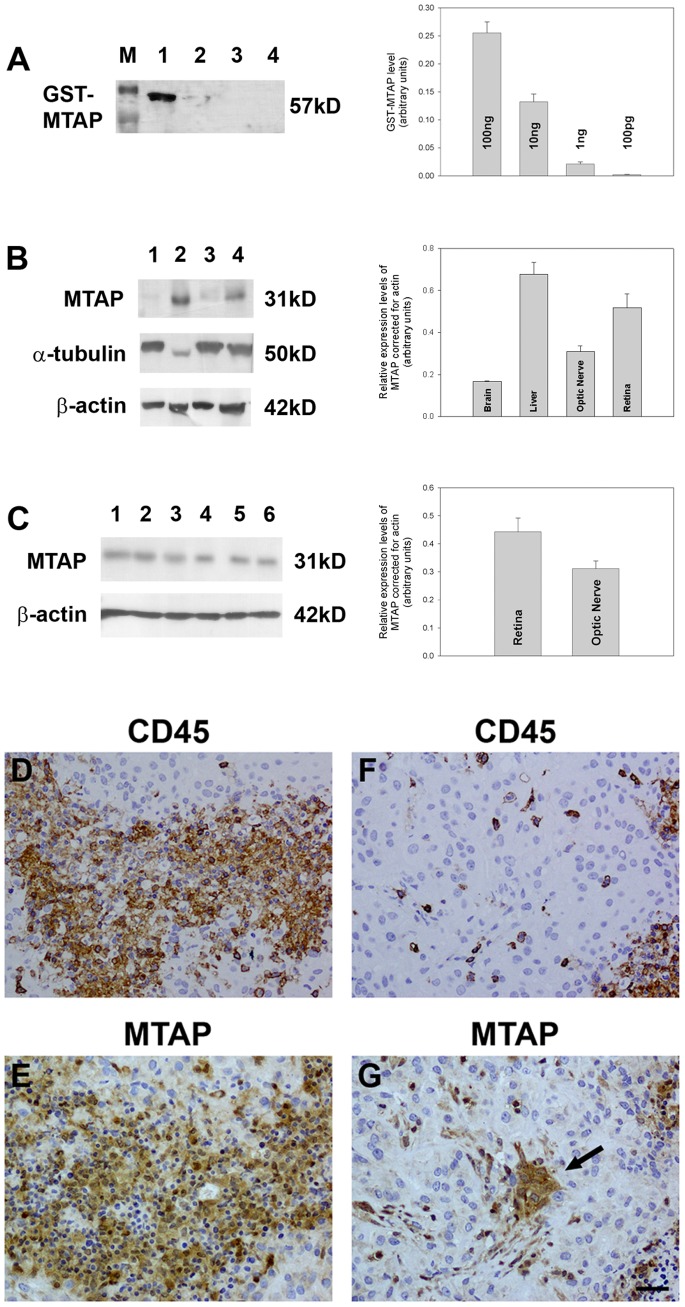
Evaluation of MTAP expression in ocular tissues. (A) Evaluation of the MTAP antibody by Western immunoblotting using GST-tagged, full length, recombinant, human MTAP protein (rMTAP). Molecular weight markers were used to determine size of detected gel products (A; lane M). Lane 1, 100 ng rMTAP; Lane 2, 10 ng rMTAP; Lane 3, 1 ng rMTAP; Lane 4, 100 pg rMTAP. A single band of the expected molecular weight (including GST-tag) is apparent; results plotted on bar graph to show quantitative distribution. (B) Rat brain cortex (lane 1), liver (lane 2), optic nerve (lane 3) and retina (lane 4) samples probed for MTAP. A protein is detected at the correct molecular mass for MTAP in the liver, optic nerve and retinal samples. Labelling for β-actin (housekeeping gene) and α-tubulin (neuronal tissue marker) are also shown; results plotted on bar graph to show quantitative distribution. (C) Evaluation of MTAP expression in retina (lanes 1–3) and optic nerve (lanes 4–6) samples obtained from three different rats. MTAP protein is detectable in all samples; results plotted on bar graph to show quantitative distribution. (D–G) Evaluation of MTAP antibodies in malignant pleural mesothelioma. In formalin-fixed, paraffin-embedded tissue sections, many inflammatory cells, as identified by immunoreactivity for CD45 (leukocyte common antigen; D), display positive labelling for MTAP using the Proteintech antibody (PT Ab; E). Some fibroblastic cells, negative for CD45 (F), also display positive labelling for MTAP using the PT antibody (G, arrow). Scale bar = 30 µm.

To investigate ocular expression of MTAP protein, three distinct commercially available antibodies (CST, Santa-Cruz, Proteintech) were obtained. Initially, each antibody was tested for reactivity against recombinant human MTAP (rMTAP), which, when conjugated to GST, has a predicted molecular mass of 57 kD. All three antibodies were able to detect 100 ng rMTAP (figure 2A); the Proteintech and Santa-Cruz antibodies also detected 10 ng rMTAP (Fig. S1A). When incubated with rat tissue extracts, all three antibodies were able to distinguish proteins at the predicted molecular mass (31 kD) in liver and retina, but only the Proteintech and Santa-Cruz preparations detected a signal in the optic nerve (Fig. 2, S1B).

Previous work has used immunohistochemistry to reliably detect MTAP expression in formalin-fixed, paraffin-embedded malignant pleural mesothelioma [37]. As a consequence, such tissue served as a positive control in the present study, both in terms of analyzing the sensitivity of the various primary antibodies used and in verifying the effectiveness of our immunohistochemistry methodology. The MTAP antibody from Proteintech correctly identified inflammatory cells and fibroblasts with a good signal-to background ratio (Fig. 2D–G). This finding, coupled with the positive results obtained from the Western blotting experiments, attested to its reliability in detecting MTAP. In contrast, the antibodies from CST and Santa-Cruz yielded weaker patterns of immunolabelling than that obtained with the Proteintech antibody (Fig. S2), even at low antibody dilutions. This result was replicated when the antibodies were tested on ocular sections, hence only data obtained using the Proteintech antibody are shown hereafter.

#### Ocular expression of MTAP

**Figure 3:**
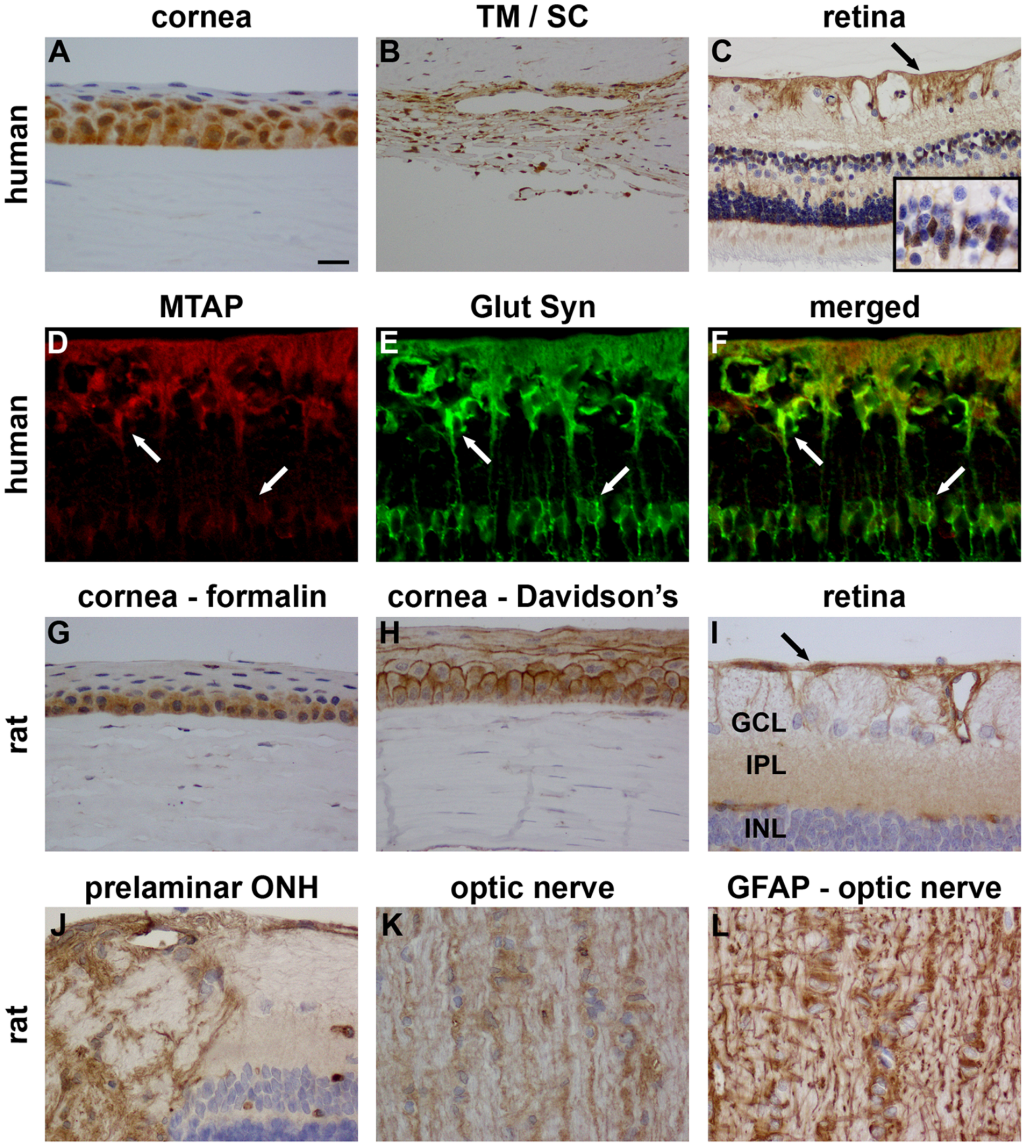
Representative images of MTAP immunolabelling in human (A–F) and rat (G–L) ocular tissues. In formalin-fixed, paraffin-embedded human eyes, positive labelling for MTAP is discernible in cells located in the corneal epithelium (A) and trabecular meshwork/Schlemm’s canal (B). In the retina, MTAP labelling is associated with astrocytes (arrow) and Müller cell somas (inset) and processes (C). Verification of MTAP expression by Müller cells was obtained by double-labelling immunofluorescence of MTAP (red) with the Müller cell marker glutamine synthetase (Glut Syn; green; D–F). In formalin-fixed, paraffin-embedded rat eyes positive labelling for MTAP is similarly detectable in corneal epithelial cells (G). Interestingly, in eyes fixed in Davidson’s solution, which affords superior morphological preservation, MTAP expression is strongly detected within the membranes of corneal epithelial cells (H). In the retina (I), ONH (J) and ON (K), MTAP labelling is principally localised to astrocytes. The pattern of immunoreactivity displays marked similarity to GFAP, as illustrated in (L). Scale bar: A, D–L = 15 µm; B, C = 30 µm. TM, trabecular meshwork; SC, Schlemm’s canal; GCL, ganglion cell layer; IPL, inner plexiform layer; INL, inner nuclear layer; ONH, optic nerve head.

In human eye sections, MTAP was associated with cells in the corneal epithelium, trabecular meshwork and retina (Fig. 3A–C). Further investigation of the localisation within the retina, which was achieved by double-labelling immunofluorescence with glutamine synthetase, revealed MTAP immunoreactivity to be principally associated with astrocytes and Müller cells (Fig. 3D–F). An association with neuronal components cannot be ruled out, however. MTAP displayed a broadly similar pattern of immunolabelling in rat ocular sections to that observed in humans (Fig. 3-L). Of note, however, in the retina MTAP appeared to be restricted to astrocytes. Unlike the human eye, no labelling of Müller cell somas or processes was apparent.

Analysis of retina and optic nerve samples from three rats revealed MTAP expression in all samples (Fig. 2C). Densitometry indicated that although there was a tendency for there to be a higher expressed level of the MTAP protein in the retina versus the optic nerve, this was not statistically significant (Fig. 2C).

### P16^INK4A^ (CDKN2A)

#### RT-PCR

P16^INK4A^ and p19^ARF^ are both products of the rat *CDKN2A* gene, each having a unique first exon, but sharing exons 2 and 3. In preliminary results, contained within our previous study [7], we made use of primers that amplified a cDNA region common to both mRNAs. Herein, we have again used these primers for comparative purposes, but also primers specific to each transcript. Table 4 shows the expression level of p16^INK4A^ mRNA, relative to the housekeeping gene cyclophilin, in rat brain cortex, liver, optic nerve and retina. The common primers detected a very low level of expression of p16^INK4A^/p19^ARF^ in the brain, retina and liver, but a greater amount within optic nerve samples. Using the p16^INK4A^-specific primers, a very low level of expression was measured in all four tissues, which, in the retina and optic nerve, approximated to 0.001% and 0.006%, respectively, of the level of cyclophilin.

**Table 4:** Expression of P16^INK4A^/P19^ARF^ mRNA in rat tissues.

		retina	optic nerve	brain	liver
cyclophilin	C_T_	17.6±0.1	19.5±0.2	18.2±0.1	19.5±0.2
P16^INK4A^/	C_T_	32.1±0.4	27.1±0.3	33.8±0.2	32.4±0.4
P19^ARF^	1ΔC_T_	14.5±0.4	7.6±0.2	15.6±0.1	12.9±0.2
	2mRNA level	8×10^−5^±2×10^−5^	0.007±0.001	3×10^−5^±3×10^−6^	2×10^−4^±0.002
P16^INK4A^	C_T_	34.7±0.1	35.4±0.3	36.8±0.4	37.3±0.0
	1ΔC_T_	17.2±0.1	15.0±0.3	18.6±0.3	17.8±0.4
	2mRNA level	1×10^−5^±1×10^−6^	6×10^−5^±2×10^−5^	4×10^−6^±1×10^−6^	1×10^−5^±1×10^−6^
P19^ARF^	C_T_	32.6±0.5	28.9±0.2	33.8±0.1	35.0±0.4
	1ΔC_T_	15.0±0.5	9.4±0.1	15.6±0.0	15.5±0.4
	2mRNA level	7×10^−5^±3×10^−5^	0.002±3×10^−4^	3×10^−5^±1×10^−6^	3×10^−5^±1×10^−6^

C_T_, cycle threshold;

1 ΔC_T_, target C_T_ - cyclophilin C_T_;

2 target mRNA level expressed relative to cyclophilin.

#### Antibody validation

**Figure 4:**
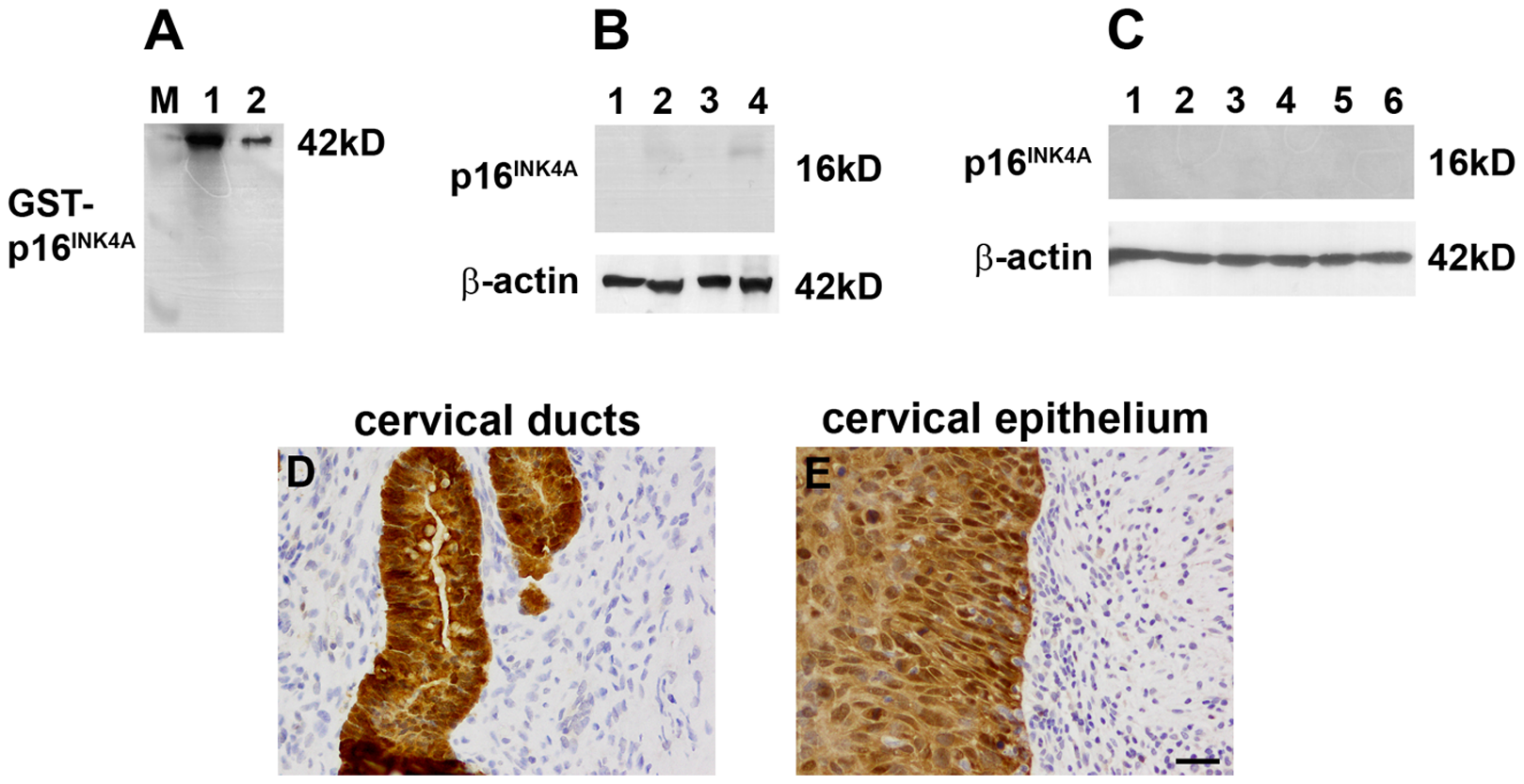
Validation of the p16^INK4A^ antibody from Santa-Cruz (sc-1661). (A) Evaluation of the specificity of the p16^INK4A^ antibody, sc-1661, by Western blotting, using GST-tagged, full length, recombinant, human p16^INK4A^ protein (rp16^INK4A^). Molecular weight markers were used to determine size of detected gel products (A; lane M). Lane 1, 100 ng of rp16^INK4A^; Lane 2, 10 ng rp16^INK4A^. A single band of the expected molecular weight (including GST-tag) is apparent. (B) Rat brain cortex (lane 1), liver (lane 2), optic nerve (lane 3) and retina (lane 4) samples probed for p16^INK4A^ protein, using sc-1661. None of the four tissues under investigation unambiguously express p16^INK4A^ protein. Labelling for β-actin (house-keeping gene product) is also shown. (C) The antibody was further tested against retina (lanes 1–3) and optic nerve (lanes 4–6) samples obtained from three different rats. The presence of p16^INK4A^ protein is not detectable in any sample. (D, E) In formalin-fixed, paraffin-embedded tissue sections of cervical adenocarcinoma, incubation with sc-1661, identifies so-called ‘block’ or ‘diffuse’ immunolabelling of ductal (D) and epithelial (E) tissue with a high signal-to-background, as compared to adjacent tissue (arrow). Scale bar = 30 µm.

For dissemination of expression levels of the p16^INK4A^ protein in ocular tissues, four antibodies (Santa-Cruz, Proteintech, Sigma and Abnova) were investigated (Figs. S3, S4; see also Fig. 4). Full length GST-tagged recombinant p16^INK4A^ protein was initially used as a positive control to determine antibody reactivity (Fig. S3A). The Proteintech, Sigma and Santa-Cruz (see also Fig. 4A) antibodies detected the recombinant protein at the correct molecular mass; the Abnova antibody did not recognise any protein signal. None of the antibodies reacted against recombinant p15^INK4B^ protein (data not shown). Subsequent analysis of the antibodies in rat brain, liver, optic nerve and retina determined that none of the four tissues under investigation expressed measureable amounts of p16^INK4A^ protein (Fig. S3B; see also Fig. 4B). Overexpression of p16^INK4A^ protein is consistently encountered in cervical dysplasias, neoplasias or carcinomas of squamous and glandular cell/tissue types that are associated with high-risk human papillomavirus infection. Indeed, immunohistochemistry for p16^INK4A^ is currently the subject of much attention regarding its suitability as a biomarker for cervical cancer screening [38], [39]. As such, formalin-fixed, paraffin-embedded tissue sections from abnormal cervical tissue represented the ideal positive control for p16^INK4A^ immunohistochemistry in the current study (Fig. S4). The antibodies from Santa-Cruz (see also Fig. 4D, E), Proteintech, and, to a somewhat lesser extent, Sigma identified p16^INK4A^-overexpressing glandular and epithelial cells with good signal-to background ratios. The p16^INK4A^ antibody from Abnova less reliably demarcated abnormal tissue and elicited robust nuclear labelling of cells in surrounding tissue. The combined data raise serious questions about the specificity of the Abnova antibody, but indicate that data obtained from the other antibodies are valid.

#### Ocular expression of p16^INK4A^

**Figure 5:**
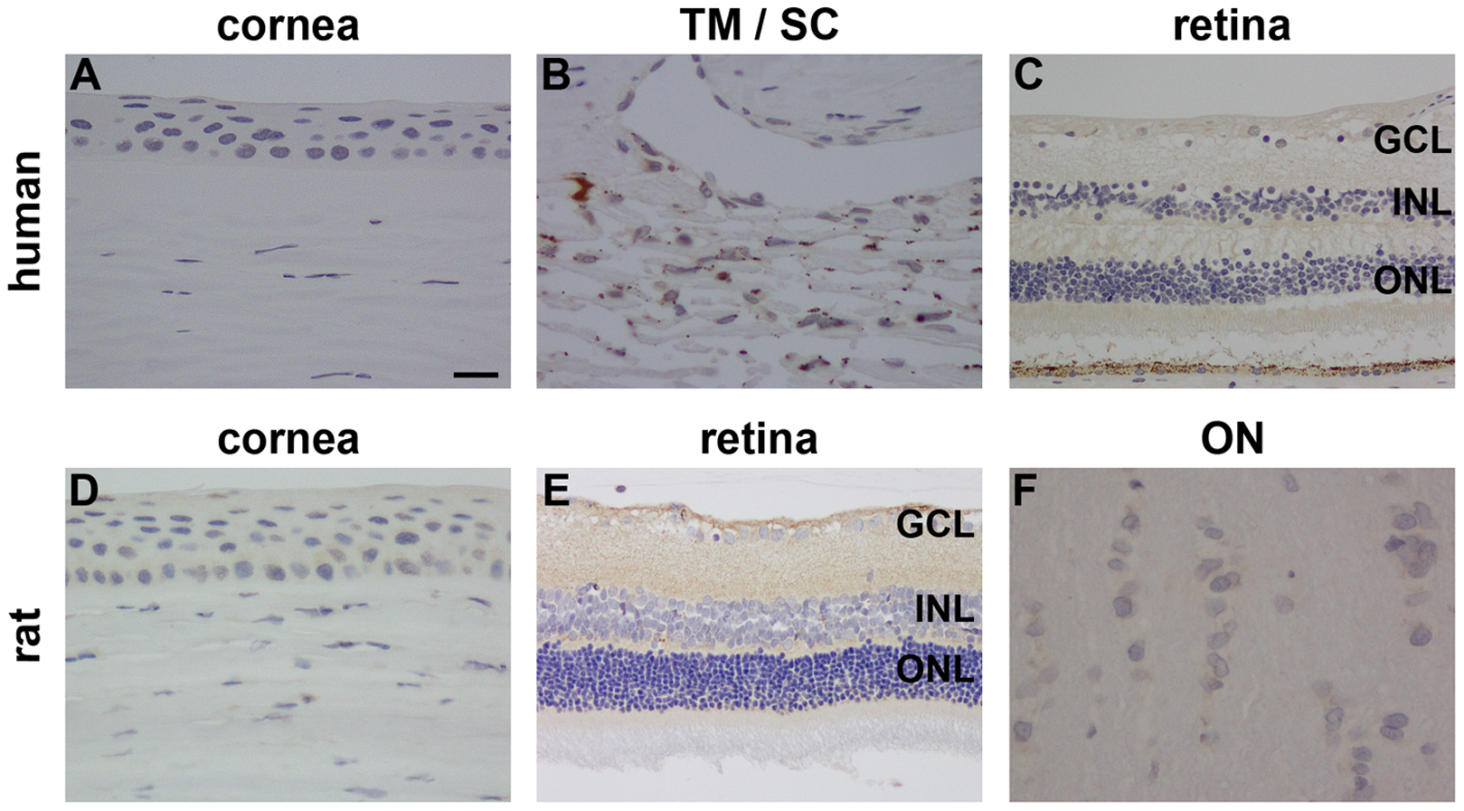
Representative images of p16^INK4A^ immunolabelling in human and rat ocular tissues. In formalin-fixed, paraffin-embedded human eyes, no positive labelling for p16^INK4A^ is discernible in the corneal epithelium (A), trabecular meshwork (TM)/Schlemm’s canal (SC) (B), or retina (C). In formalin-fixed, paraffin-embedded rat eyes, no positive labelling for p16^INK4A^ is discernible in the corneal epithelium (D), retina (E) or optic nerve (F). Scale bar: A, B, D, F = 15 µm; C, E = 30 µm. GCL, ganglion cell layer; INL, inner nuclear layer; ONL, outer nuclear layer.

In human eye sections, incubated identically to and simultaneously with cervical tissue sections, no specific p16^INK4A^ immunolabelling was observed in the cornea, drainage angle, retina or optic nerve (Fig. 5A, B, C; see also Fig. S5); note the presence of pigment and not positive immunolabelling in the TM/Schlemm’s Canal images (Fig. 5B). More concentrated dilutions simply resulted in generic, non-specific staining (data not shown). Similarly, in rat ocular sections, no unambiguous evidence was found for p16^INK4A^ protein expression in the cornea, retina or optic nerve (Fig. 5D, E, F; see also Fig. S6). In agreement with these findings, Western immunoblotting analysis of retina and optic nerve from three rats revealed no expression of p16^INK4A^ in any sample (Fig. 4C; see also Fig. S3C).

### P14^ARF^

#### Antibody validation

**Figure 6:**
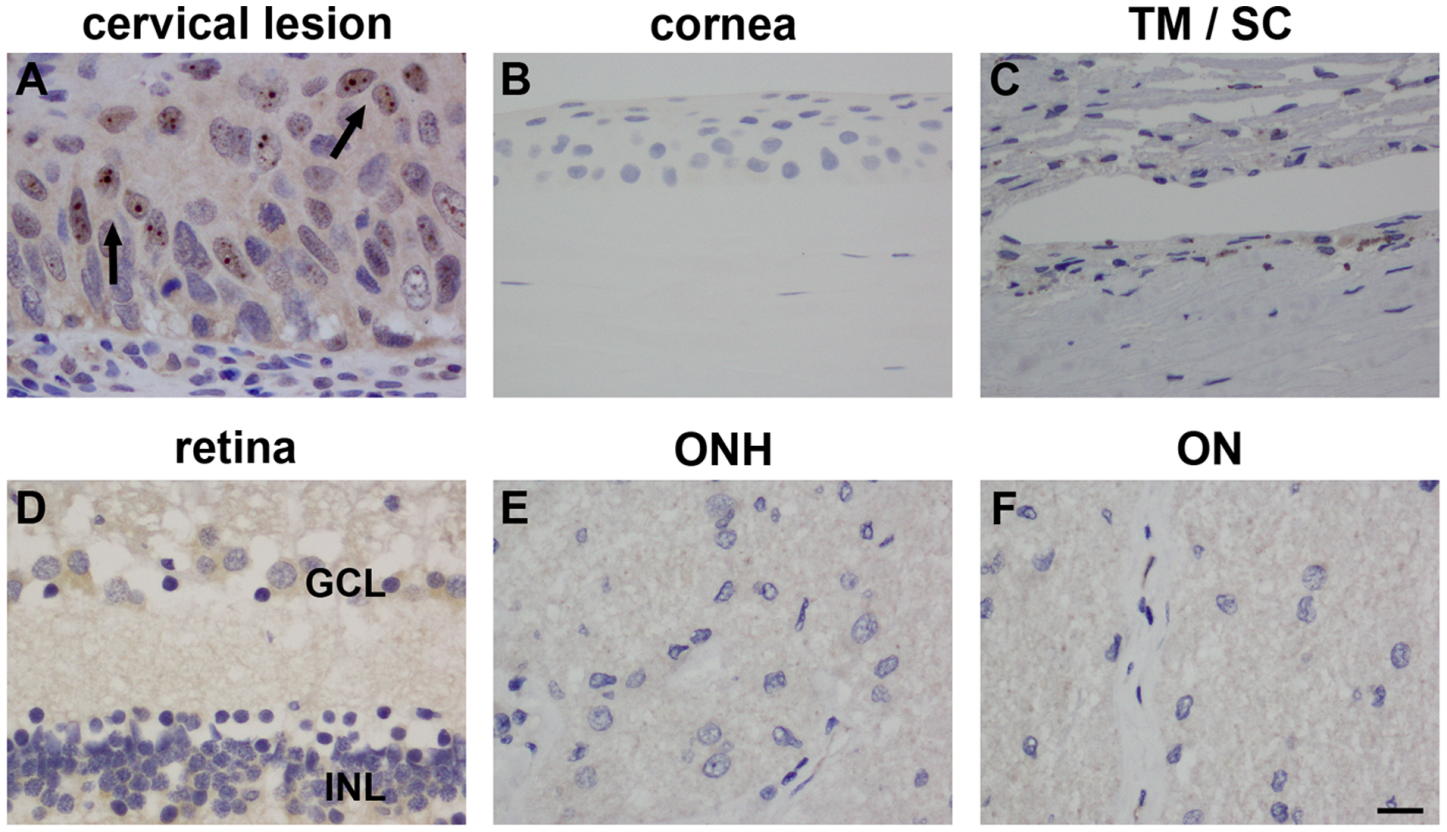
Representative images of p14^ARF^ immunolabelling in human ocular tissues. In formalin-fixed, paraffin-embedded sections of cervical intraepithelial neoplasia, p14^ARF^ expression is identified, as expected, within nuclear inclusions (A). In formalin-fixed, paraffin-embedded human eyes, no positive labelling for p14^ARF^ is discernible in the corneal epithelium (B), trabecular meshwork (TM)/Schlemm’s canal (SC) (C), retina (D), unmyelinated optic nerve (E), or myelinated optic nerve (F). Scale bar = 15 µm. GCL, ganglion cell layer; INL, inner nuclear layer.

P14^ARF^, the human homologue of p19^ARF^, has been shown to be localized mainly in the cellular nucleolus; it is also present in the nucleoplasm of cells expressing the protein [40]. Characteristically, induction of p14^ARF^ occurs in response to inappropriate hyperproliferative signals, such as in cancerous tissues. Alongside p16^INK4A^, expression of p14^ARF^ has consistently been detected in abnormal cervical tissue [40], [41]; thus, we again used formalin-fixed, paraffin-embedded tissue sections from cervical lesions as a positive control for p14^ARF^ immunohistochemistry. Both antibodies tested (Sigma and CST) identified p14^ARF^ expression in nuclear inclusions within epithelial cells in abnomal cervical tissue, with the antibody from Sigma (Fig. 6A) displaying a better signal-to background ratio than the antibody from CST (Fig. S7A).

#### Ocular expression of p14^ARF^

In human eye sections, incubated identically to and simultaneously with cervical tissue sections, neither antibody revealed any specific, nuclear p14^ARF^ immunolabelling in the cornea, drainage angle, retina or optic nerve (Fig. 6B, C, D, E, F; Fig. S7).

### P19^ARF^

#### RT-PCR

Table 4 shows the expression level of p19^ARF^ mRNA, relative to the housekeeping gene cyclophilin, in rat brain, liver, optic nerve and retina. As noted above, the common primers detected a very low level of expression of p16^INK4A^/p19^ARF^ in the brain, retina and liver, but a greater amount within optic nerve samples. Using the p19^ARF^-specific primers, it was confirmed that the levels of transcript in the brain, liver and retina were indeed low (approximately 0.003–0.007% of the level of cyclophilin), and, that the greater amount in the optic nerve (approximately 0.2% of the level of cyclophilin) represented p19^ARF^ rather than p16^INK4A^.

#### Antibody validation and ocular expression

**Figure 7:**
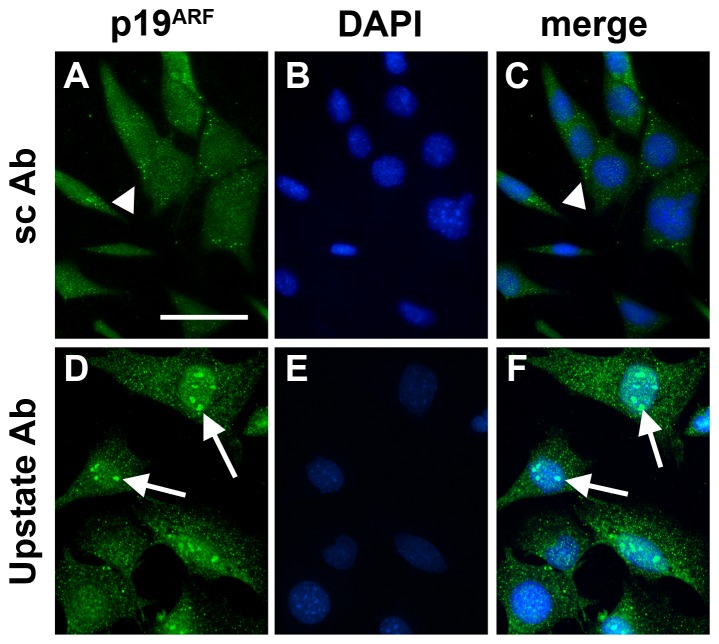
Immunocytochemical localization of p19^ARF^ protein in cultured, undifferentiated, non-confluent, rodent RGC-5 cells. (A–C) Use of the Santa Cruz antibody (A), shown in relation to nuclear DAPI counterstaining (B, C), reveals little P19^ARF^ protein labeling except for some cytoplasmic granules (arrowheads). There is no obvious nuclear labeling. (D–F) Use of the p19^ARF^ antibody from Upstate, however, denotes clear nuclear labeling within cells. The positive labeling appears to be present in nucleolar-like structures within cell nuclei (as denoted by DAPI-positive staining; arrows). This labeling is present in approximately 75% of cells. Scale bar = 10 µm.

Expression of p19^ARF^ protein in rat ocular tissues was examined using two distinct, commercially-available antibodies (Upstate, Santa-Cruz). Initially, to visualise positive immunolabelling for P19^ARF^, transformed, retina-derived, undifferentiated, rodent RGC-5 cells were grown to confluence and stained with both antibodies. The rationale for this test is that previous work has demonstrated p19^ARF^ localization in the nucleolus of immortalised cells [42]. The Santa-Cruz antibody appeared to label only some cytoplasmic granules (Fig. 7A, B, C), but the Upstate antibody clearly labelled nuclear inclusions (Fig. 7D, E, F). Since the immunocytochemical labelling in RGC-5 cells with the Upstate antibody resembled that reported previously for other immortalised cells [42], we believe this to be specific labelling and therefore the most applicable antibody for future studies.

**Figure 8:**
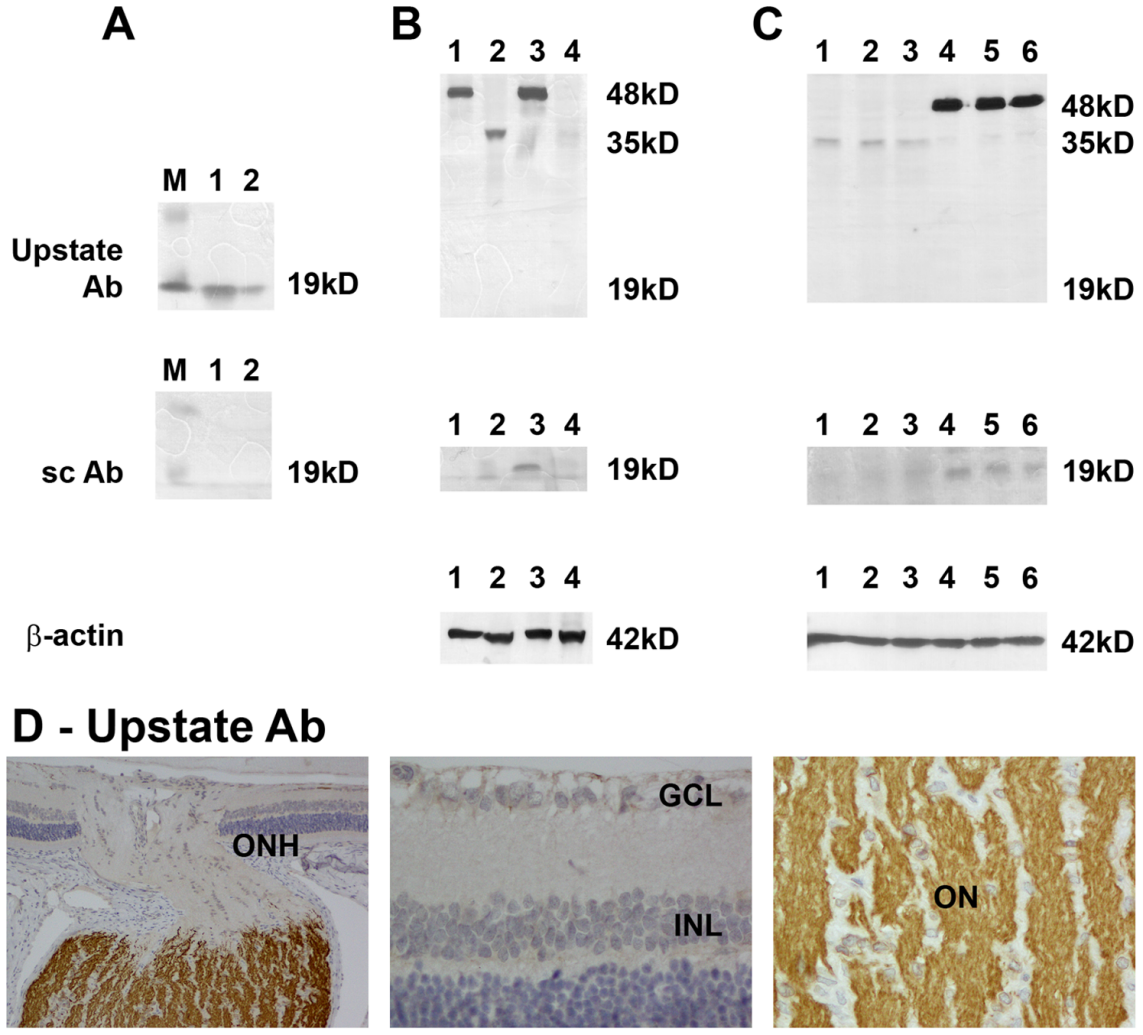
Evaluation of p19^ARF^ antibodies, and p19^ARF^ expression in rat retina and optic nerve. (A) Evaluation of two p19^ARF^ antibodies, from Upstate and Santa-Cruz (sc), by Western immunoblotting using full length, recombinant rat p19ARF protein (r P19^ARF^). For each antibody tested, molecular weight markers were used to determine size of detected gel products (A, C; lane M). Lane 1, 100 ng of r P19^ARF^; Lane 2, 10 ng rP19^ARF^. Single bands of the correct molecular mass are detectable by the Upstate antibody, but not by the Santa Cruz antibody. (B) Rat brain cortex (lane 1), liver (lane 2), optic nerve (lane 3) and retina (lane 4) samples probed for p19^ARF^ protein with the Upstate and sc antibodies. The Upstate antibody does not recognize any proteins at the correct molecular mass, but the sc antibody recognises a 19 kD protein in the optic nerve sample. Labelling for β-actin (house-keeping gene product) is also shown. (C) Further testing of both antibodies against retina (lanes 1–3) and optic nerve (lanes 4–6) samples obtained from three different rats reveals that the Upstate antibody fails to detect a band at 19 kD in any sample, but detects a protein of approximately 38 kD in the retinal, and a protein of 48 kD in the optic nerve, samples. The sc antibody recognises a 19 kD protein in each optic nerve sample but not in the retinal samples. (D) Representative images of p19ARF immunolabelling using the Upstate p19^ARF^ antibody in the rat retina and optic nerve. No nuclear staining is detectable in cells of the retina or optic nerve; however, robust labelling of axons in the myelinated, but not unmyelinated, optic nerve is apparent. This is most clearly shown in the image taken of the optic nerve head (ONH). Scale bar = 30 µm. GCL, ganglion cell layer; INL, inner nuclear layer.

To further verify the specificity of the Upstate and Santa-Cruz antibodies, both preparations were tested against recombinant rat p19^ARF^ by Western blotting. The Upstate antibody clearly detected 100 ng and 10 ng recombinant p19^ARF^, but the Santa-Cruz antibody was unable to distinguish any signal (Fig. 8A). When analyzing rat tissue samples, however, the Upstate antibody failed to detect any proteins at the correct molecular mass (19 kD). Moreover, in brain cortex and optic nerve samples a protein with a mass of 48 kD was detected, and in liver and retina samples a protein with the mass of 35 kD was weakly distinguished (Fig. 8B). This was confirmed when 3 independent retina and optic nerve samples were analysed (Fig. 8C). From these results, it appears likely that the Upstate antibody cross-reacts with other proteins. Surprisingly, since it did not cross-react with the recombinant rat p19^ARF^, the Santa-Cruz antibody detected a 19 kD protein in optic nerve but not retina, brain cortex or liver samples (Figs. 8B, C).

#### Immunohistochemistry

In rat ocular sections, no evidence was found for nuclear p19^ARF^ protein expression in the anterior segment (data not shown), retina (Fig. 8D) or optic nerve (Fig. 8D) when using the Upstate antibody. Interestingly, sections through the rat optic nerve head demonstrated a cross-reaction of the antibody with the myelinated portion of the optic nerve (Fig. 8D). This quite likely accounts for the non-specific band at 48kD observed in Western blots. As regards the Santa-Cruz antibody, no nuclear p19^ARF^ protein expression was observed in the anterior segment, retina or optic nerve (data not shown).

The overall results for p19^ARF^ suggest that the protein is not present at detectable levels in rat ocular tissues; however, the profile of neither antibody is totally convincing and some doubt remains.

### P15^INK4B^ (CDKN2B)

#### RT-PCR

Table 5 shows the expression level of p15^INK4B^ mRNA, relative to the housekeeping gene cyclophilin, in rat brain, liver, optic nerve and retina. The p15^INK4B^ transcript is present in considerably higher amounts in the optic nerve than in the retina (approximately 1.8% and 0.02% of the level of cyclophilin, respectively). Of interest is the fact that in both tissues expression was markedly higher than for p16^INK4A^. The levels of p15^INK4B^ mRNA in the brain and liver were broadly similar to that measured in the retinal samples.

**Table 5:** Expression of mRNA for p15^INK4B^ in rat tissues.

		retina	optic nerve	brain	liver
cyclophilin	C_T_	17.6±0.1	19.5±0.2	18.2±0.1	19.5±0.2
p15^INK4B^	C_T_	31.0±0.3	25.6±0.1	31.7±0.1	30.6±0.4
	1ΔC_T_	13.3±0.3	6.0±0.2	13.5±0.1	11.1±0.1
	2mRNA level	0.0002±3×10^−5^	0.018±0.001	0.0001±1×10^−5^	0.0063±0.0002

C_T_, cycle threshold;

1 ΔC_T_, target C_T_ - cyclophilin C_T_;

2 target mRNA level expressed relative to cyclophilin.

#### Antibody validation

To determine expression of p15^INK4B^ in rat ocular tissues, four distinct, commercially available antibodies (CST; Neomarkers; Abcam; Santa-Cruz) were employed. Initially, each antibody was tested against recombinant rat p15^INK4B^ by Western blotting. Next, each antibody was tested against a variety of rat tissues by Western blotting. For the purposes of immunohistochemistry, antibodies were validated using formalin-fixed, paraffin-embedded tissue sections from abnormal cervical tissue and normal skin, since p15^INK4B^ positive immunolabelling has previously been detected in cervical intraepithelial neoplastic lesions [43] and in cutaneous squamous cells [44].

**Figure 9:**
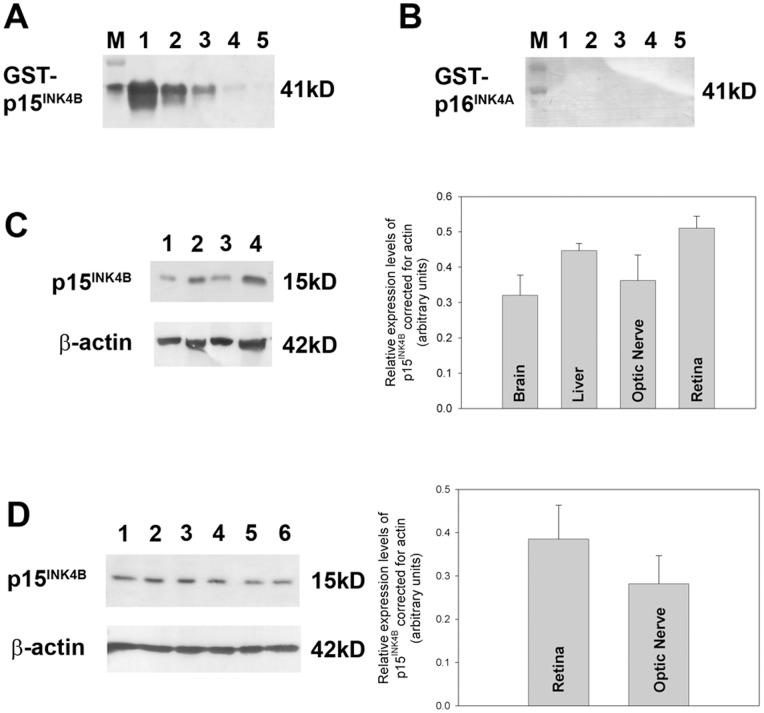
Evaluation of p15^INK4B^ expression by Western immunoblotting. (A, B) Validation of p15^INK4B^ antibody specificity by Western blotting using GST-tagged, full length, recombinant human p15^INK4B^ (rp15^INK4B^) and p16^INK4A^ (rp16^INK4A^) proteins. M, molecular weight markers. (A) Reactivity of antibody to rp15^INK4B^, where lane 1, 100ng of rp15^INK4B^; Lane 2, 10 ng rp15^INK4B^; Lane 3, 1 ng rp15^INK4B^ P; Lane 4, 100 pg rp15^INK4B^. Single bands of the expected molecular weights (for protein incorporating GST-tag) are apparent. (B) Reactivity of antibody to rp16^INK4A^ in order to determine any non-specific binding to the highly related protein p16^INK4A^, where lane 1, 100 ng of rp16^INK4A^; Lane 2, 10 ng rp16^INK4A^; Lane 3, 1 ng rp16^INK4A^; Lane 4, 100 pg rp16^INK4A^. (C) Rat brain cortex (lane 1), liver (lane 2), optic nerve (lane 3) and retina (lane 4) samples probed for rp15^INK4B^. A 15 kD protein is detectable in all tissue samples; results plotted on bar graph to show quantitative distribution. (D) Expression of p15^INK4B^ in retinal (lanes 1–3) and optic nerve (lanes 4–6) samples. Labelling for β-actin (house-keeping gene product) is also shown; results for p15^INK4B^ plotted on bar graph to show quantitiative distribution, relative to actin levels.

**Figure 10:**
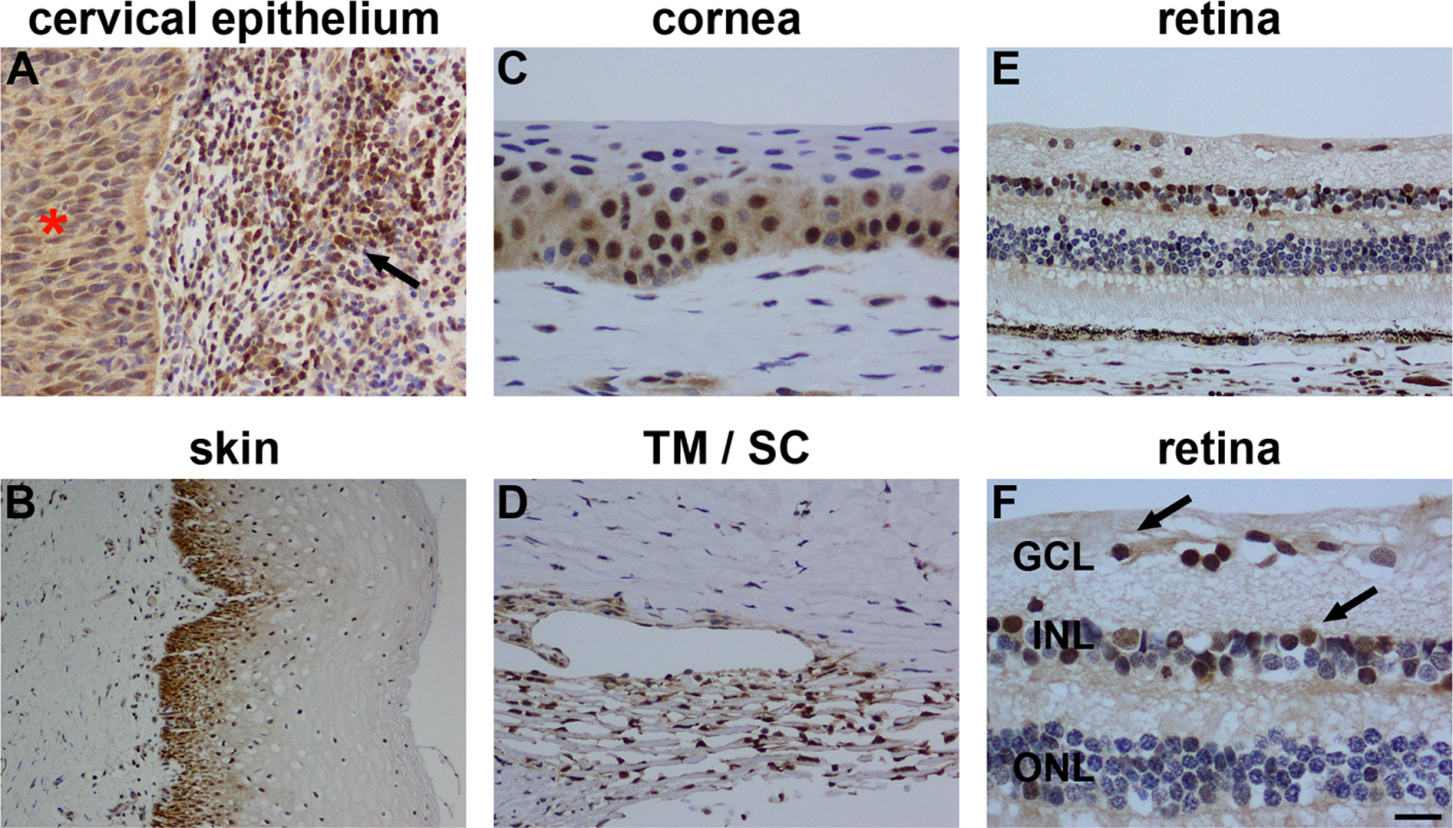
Representative images of p15^INK4B^ immunolabelling in human tissues. In formalin-fixed, paraffin-embedded tissue sections, weak, diffuse p15^INK4B^ immunolabelling of the cervical intraepithelial neoplastic lesion (red asterisk) and robust labelling of a subset of inflammatory cells in the underlying stroma (arrow) is observed (A). In skin sections, p15^INK4B^ immunoreactivity is associated with cutaneous squamous cells (B, arrows). In formalin-fixed, paraffin-embedded human eyes, positive labelling for P15^INK4B^ is discernible in the nuclei of cells located in the corneal epithelium (C) and trabecular meshwork/Schlemm’s canal (D). In the retina, P15^INK4B^ labelling is associated with the nuclei of cells within the GCL and INL (arrows; E, F). Scale bar: C, F = 15 µm; A, D, E = 30 µm; B = 60 µm. GCL, ganglion cell layer; INL, inner nuclear layer.

The p15^INK4B^ antibody from CST was able to detect the correct mass protein (GST-p15INK4B; 41 kD) when present at 100 pg-100 ng (Fig. 9A), but importantly did not cross-react with recombinant GST-p16^INK4A^ (Fig. 9B). When tested against a variety of rat tissues by Western blotting, the CST antibody correctly recognised 15 kD products: in particular, positive reactivity was detected in liver (as defined previously [45]), retina and optic nerve (Fig. 9C). The specificity of the CST antibody was confirmed in additional retina and optic nerve samples (Fig. 9D), where no significant differences were found in expression levels. In tissue sections from abnormal cervical tissue (Fig. 10A) and normal skin (Fig. 10B), the CST antibody yielded patterns of immunolabelling in agreement with those previously described [43], [44]. These results attest to the validity of the CST antibody for recognition of the p15^INK4B^ protein. In contrast, the p15^INK4B^ antibodies from Neomarkers, Abcam and Santa-Cruz produced unsatisfactory results (see Figs. S8, S9).

#### Ocular expression of p15^INK4B^

**Figure 11:**
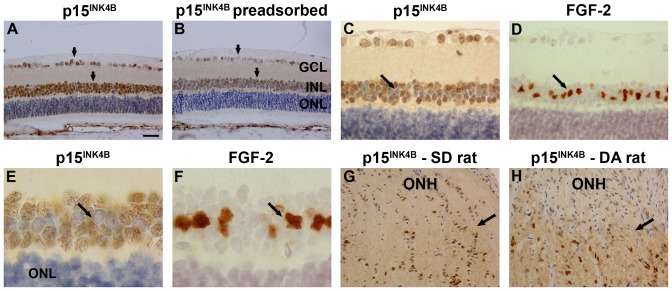
Representative images of p15^INK4B^ expression in rat retina and optic nerve. (A) P15^INK4B^ immunoreactivity is associated with nuclei within the GCL and INL (arrows), the intensity of which is substantially reduced after preadsorption with recombinant P15^INK4B^ protein (B). Greater magnification images of retinas labelled with the CST antibody (C, E) reveal a pattern of labelling in the INL that encompasses neurons, but not Müller cells. For comparative purposes, the distribution of Müller cells is revealed in adjacent sections using an antibody directed against FGF-2 (D, F). In the optic nerve, labelling is observed within the nuclei of a subset of glial cells. Labelling is stronger in the myelinated portion of the nerve than in the unmyelinated ONH (black arrow indicates myelination transition zone). This effect is more pronounced in the Dark-Agouti (DA; H) strain of rat than the Sprague-Dawley strain (SD; G). Scale bar: A, B, G, H = 30 µm; C, D = 15 µm; E, F = 6 µm. GCL, ganglion cell layer; INL, inner nuclear layer; ONL, outer nuclear layer; FGF-2, fibroblast growth factor-2; ONH, optic nerve head.

In human eye sections, p15^INK4B^ immunolabelling, as identified by use of the CST antibody, was associated with the nuclei of cells located in the corneal epithelium, trabecular meshwork and inner retina (Fig. 10C, D, E, F). In rat eye sections, p15^INK4B^ immunolabelling was associated with nuclei in the ganglion cell and inner nuclear layers, but not in the outer nuclear layer (Fig. 11A). Preadsorption with recombinant p15^INK4B^ protein reduced the intensity of labelling (Fig. 11B). More detailed scrutiny of the pattern of labelling in the retina revealed that it encompassed neurons but not Müller cells (Fig. 11C, D, E, F). In the optic nerve, p15^INK4B^ immunolabelling was associated with the nuclei of glial columns (Fig. 11 G, H), particularly in the myelinated portion of the nerve.

## Discussion

In light of previous work defining the importance of gene products from the 9p21 locus in diseases such as coronary heart disease [21], [22], [23], diabetes [21], [46], atherosclerosis [26] and cancer [24], [25], the systematically validated discovery that SNPs from this region are also associated with POAG is of great significance [7], [8], [11], [12], [13], [14], [15], [16], [17], [18], [19], [20]. The logical first step in disseminating the role that such gene products might play in POAG is an investigation into their expression and localization within the normal eye; such was the purpose of the current study. In undertaking this project, we concentrated on both human and rat ocular tissues. Rodent models of glaucoma are increasingly used to investigate the pathogenesis of retinal ganglion cell loss; thus, delineation of the expression patterns in both species permits a better understanding of the commonality between humans and rats and the relevance of conducting basic research.

Owing to the absence of suitable tissue from human eyes for analysis by Western blotting and RT-PCR, these latter techniques were restricted to the rat. This situation inevitably resulted in the main focus of the current study being on visualisation of 9p21 gene products by immunohistochemistry. This is not necessarily a straightforward task, despite the proliferation of commercially available antibodies, since biologically meaningful immunohistochemical data can only be obtained if the antibodies in question are meticulously validated using the methodologies employed by the researchers. Disturbingly, commercial antibodies often fail rudimentary tests of specificity [47], which, when combined with inadequate controls, leads to publication of erroneous results. The interrelated topics of antibody specificity and appropriate controls for use with immunohistochemistry have been covered in numerous reviews and editorials in recent years [47], [48], [49], [50]. A number of immunohistochemistry controls can be employed, of which some of the more useful are as follows: (1) Western blot data showing both that the primary antibody can bind specifically to a correctly sized antigen and that such a band is observed in tissues displaying a positive immunohistochemical signal; (2) lack of signal in tissue from a knockout animal; (3) comparison of results from separate antibodies raised against different epitopes of the protein; (4) lack of signal in tissue with a known minimal level of expression of the molecule; (5) presence of cell-type specific labelling at high signal-to-background in tissue with known expression of the molecule; (6) correlation of protein expression with mRNA expression, for example by in situ hybridization or RT-PCR; (7) absence of signal when the primary antibody is preincubated with an excess of the antigen prior to use (preadsorption test). With the exception of the relevant knockout rats, which we were unable to obtain for this investigation, we have made use of all of these controls.

### MTAP

MTAP, an enzyme that plays an essential role in the salvage of cellular adenine and methionine following biosynthesis of polyamines, has been shown to be present in all normal mammalian tissues analysed with particularly high levels of activity described in the liver and lung [51], [52]. Despite the growing interest in the role of MTAP, the cellular distribution of the protein in normal CNS tissue, and the eye in particular, has received negligible attention. Our results show that the levels of MTAP mRNA expression in the normal rat retina and optic nerve are comparable, each equating to about 0.3% and 1%, respectively, of the levels of the housekeeping genes glyceraldehyde 3-phosphate dehydrogenase and cyclophilin. The MTAP transcript, whilst not abundant, is expressed at a considerably higher level in the retina than the transcripts encoding p16^INK4A^, p15^INK4B^ and p19^ARF^. As regards the protein, three commercially available antibodies were tested. Although each of the antibodies detected recombinant MTAP and were able to distinguish proteins at the predicted molecular mass in liver and retina samples using Western blotting, the polyclonal antibody from Proteintech proved best suited to detection of the protein in tissue sections. This antibody has previously been validated for detection of MTAP in malignant pleural mesothelioma [37], gastro-intestinal tumors [53], [54] and chordoma [55] providing further evidence of its specificity. In the anterior segment of the human eye, positive immunolabelling was evident in both the corneal epithelium and trabecular meshwork, while in the retina, MTAP immunoreactivity was principally associated with astrocytes and Müller cells. In the rat retina, MTAP appeared to be associated with astrocytes but not Müller cells. It is possible that neuronal cells in the human and rat retina also express MTAP, but the protein abundance is below the detection limits of the immunohistochemical assay. Overall, the immunohistochemistry, Western blotting and real-time RT-PCR methodologies provided unambiguous results that correlated satisfactorily. Further studies should investigate whether MTAP expression in the trabecular meshwork and in glial cells of the retina and optic nerve is important in the pathogenesis of POAG.

### P16^INK4A^

P16^INK4A^ is a principle member of the INK4 family of proteins, which also includes p15^INK4B^, p18^INK4C^ and p19^INK4D^, and whose major function is to inhibit cyclin-dependent kinases (CDKs) and therefore exert control on the cell cycle (see review by Romagosa et al. [56]). P16^Ink4A^ itself contributes to regulation of the cell cycle by binding to CDK4 and CDK6. In the absence or malfunctioning of p16^Ink4A^, these enzymes associate with cyclin-D and phosphorylate the retinoblastoma protein, ultimately activating transcription factors of the E2F family and promoting G1/S transition. Aberrant functioning of p16^Ink4A^ can, therefore, have profound pathological influences derived from erroneous regulation of the cell cycle and/or entry of the cell into apoptosis [57]. It is of no surprise, therefore, to find that alterations in physiological expression levels of p16^Ink4A^ are associated with many types of human disease as well as with aging and senescence [56].

Preliminary data, included within our previous study [7], indicated high expression of p16^INK4A^ in rat retina. This conclusion was based on immunohistochemical staining of tissue sections with antibody clone 2D9A12 (Abnova), which yielded high signal-to-background nuclear labelling in virtually all retinal cells. We were, therefore, surprised to discover that the rat retinal samples analysed in the present study had minimal expression of p16^INK4A^ mRNA: the transcript level approximated to 0.001% to 0.008% of the level of the housekeeping cyclophilin, depending on the primer set used in the real-time PCR assay. The low level of p16^INK4A^ mRNA in the retina agrees with previous research, which has shown that p16^INK4A^ mRNA is not expressed at detectable levels in adult human [7], [58] or rodent [59], [60], [61] CNS. Although various studies have documented that p16^INK4A^ is a relatively stable protein, the mismatch between mRNA and immunohistochemistry clearly needed addressing. To test the validity of clone 2D9A12, we performed Western blotting and immunohistochemistry using three further p16^INK4A^ antibodies that are all reactive in rat and human tissue. Furthermore, we made use of arguably the definitive positive control tissue for expression of p16^INK4A^, namely abnormal cervical tissue. The diagnostic value of p16^INK4A^ overexpression has been proven in high risk human papillomavirus infections, in cervical dysplasia and in various cervical carcinomas [38], [39]. The results were clear-cut: the Santa-Cruz (sc-1661), Proteintech and Sigma antibodies all detected recombinant p16^INK4A^ on Western blots and yielded robust immunolabelling in cervical carcinoma tissue sections, but, no specific p16^INK4A^ immunolabelling was observed in the cornea, drainage angle, retina or optic nerve of human or rat tissue sections. These data correlate with the real-time PCR results and suggest that p16^INK4A^ is not expressed to any significant degree in normal human or rat ocular tissues. The results also concur with previous studies that have investigated p16^INK4A^ expression in retinoma [62] and retinoblastoma [63]. In both of these studies, one of which featured Santa-Cruz antibody sc-1661, tumor cells were typically p16^INK4A^-positive, but adjacent, healthy retina was p16^INK4A^-negative. In contrast, clone 2D9A12 gave unsatisfactory results when used for Western blotting, less reliably demarcated p16^INK4A^ overexpression in abnormal cervical cells, and, additionally elicited robust nuclear labelling of numerous cells in surrounding healthy cervical tissue in an analagous manner to that observed in ocular tissue sections. Our study raises serious questions about the specificity of Abnova clone 2D9A12. We might speculate that this antibody cross-reacts with p18^INK4C^ or p19^INK4D^, which share significant homology with p16^INK4A^ and are abundantly expressed in normal adult tissues [59], but further investigation would be needed to explore this possibility.

### P14^ARF^/P19^ARF^

Human p14^ARF^ (and the rodent equivalent, p19^ARF^) is derived from an alternative reading frame of the p16^INK4A^ gene sequence [64], [65]. Originally thought just to be a tumour-suppressor protein being co-expressed and therefore functioning in the same manner as p16^INK4A^, it was surprising to delineate that p14^ARF^ had its own distinct but complementary role in the control of cell cycle entry [66]. It is thought that this protein binds to Mdm2, which functions to ubiquitinylate the key cellular tumour-suppressor protein, p53, and therefore mark it for degradation by the proteasome [67]. When p14^ARF^ is expressed, usually under conditions of cellular stress, it sequesters Mdm2, allowing p53 levels to rise and activate the latter protein’s functions as a regulator of DNA damage repair, an abrogator of the cell cycle and a determinant in the instigation of cellular apoptosis [67]. P14^ARF^ therefore has a key role to play in the cellular response to stress and is therefore usually found, upon expression, within the nucleus.

When studying p14^ARF^ in human cancerous cervical tissue sections, this protein was localized to nuclear inclusions in epithelial cells by two distinct antibodies (CST and Sigma) in agreement with the known subcellular localization of this protein [40]. When applied to human ocular sections, however, no such labeling was seen in any cells and so we conclude that there was either no p14^ARF^ expressed in the human eye or that, if present, its levels were so low as to be undetectable by our methods.

Initially studying p19^ARF^ in rodent tissues, we found significant mRNA expression in the optic nerve relative to all other tissues examined. Subsequent analysis of tissues by labeling with two distinct antibodies (Upstate and Santa Cruz) revealed that, as expected, both labeled nuclei in immortalized mouse RGC-5 cells, with the Upstate antibody specifically recognizing nuclear inclusions which were reminiscent of nucleoli [42]. Western blot data, however, was inconsistent: the Santa Cruz antibody could not recognize the recombinant protein, but could detect a 19 kD protein in rat optic nerve extracts, whereas the Upstate antibody correctly recognized the recombinant protein, but only revealed a 48 kD protein in brain and optic nerve. When rat ocular sections were examined, the Upstate antibody revealed strong immunolabelling in the fibres of the post-laminar optic nerve. We therefore believe that this antibody was actually recognizing a different axonal protein, an idea reinforced by the positive detection of a protein in only brain and optic nerve Western extracts. These findings thus rendered this antibody as inappropriate for further study in our studies. Alongside these data, the Santa Cruz antibody could detect no signal in any ocular cell-type or region and so no conclusion could be drawn as to its eligibility for further use. The only definitive conclusion that could be drawn from the p19ARF studies, therefore, was from the mRNA analyses, which was that expression within the normal retina is negligible but within the optic nerve measurably higher.

### P15^INK4B^

Like P16^INK4A^, p15^INK4B^ is a member of the INK4 family of tumour-suppressor proteins and therefore also functions as a CDK4/6 inhibitor [68]. As with other INK4 proteins, interaction with the target CDK prevents binding of cyclin D and this in turn blocks activation of G1/S-phase transition, holding the cell in a pre-divided state [68]. Despite the similarities in the known binding properties of the INK4 group members, the specific functions of p15^INK4B^ and the reasons why two structurally and functionally similar proteins are expressed from a single gene locus remain unknown. Furthermore, specific functions of p15^INK4B^ are relatively enigmatic compared with those of p16^INK4A^. It is now believed, however, that p15^INK4B^ acts generally as a cellular back-up for p16^INK4A^ in the case of loss-of-function of the latter [69]. P15^INK4B^ has now been linked to the mechanism of TGFβ-induced cell cycle arrest [68]. This could be of relevance to glaucoma since this latter growth factor is thought to play a role in the pathogenesis of optic nerve head damage in this disease [70].

Relatively little is known about the expression patterns and roles of p15^INK4B^ in healthy mammals, but studies have shown it to be more widely distributed than p16^INK4A^ [59], [60], [71]. Our qPCR results support this generalisation: the levels of p15^INK4B^ mRNA in the rat retina and brain were broadly similar with, as expected [43], a somewhat greater amount found in the liver. In all three tissues, the p15^INK4B^ transcript was at a higher level than that of p16^INK4A^. Interestingly, our results also showed that, as was the case for p19^ARF^, there is a considerably greater expression of p15^INK4B^ mRNA in the optic nerve than in the retina, although there was no significant difference in protein levels in these tissues. Delineation of p15^INK4B^ protein expression in ocular tissues is more problematic than for p16^INK4A^, p19^ARF^ and MTAP, since, to our knowledge, there is no definitive positive control tissue that can be utilised and no antibody clone that has been well characterized. P15^INK4B^ protein expression has been documented using numerous different antibodies and the protein has been shown to be localised varyingly in the nucleus and cytoplasm. The situation is further complicated by the fact that FASTA sequence comparison of the human p15^INK4B^ and p16^INK4A^ proteins reveals 89.5% similarity, although importantly the N-termini of each protein retain little identity.

Of the four commercially available antibodies tested in the current study, two provided unsatisfactory results: (1) the Abcam p15^INK4B^ polyclonal antibody recognised recombinant p16^INK4A^ with equal potency to p15^INK4B^. This non-specific interaction obviously renders it unsuitable for further use; (2) the Santa-Cruz antibody (K-18), which has been used in numerous published studies, and, which was raised against a peptide mapping near the N-terminus engendering confidence in its specificity, provided us with expected labelling patterns in abnormal cervix and normal skin tissue sections. Unexpectedly, however, this antibody cross-reacted with an abundant 35kD protein in the retina that appears to be produced by photoreceptor outer segments. The two other antibodies used: a polyclonal antibody (CST) raised against a peptide mapping near the N-terminus and a monoclonal antibody (Neomarkers; clone 15P06) raised against full length p15^INK4B^, provided similar patterns of immunolabelling in ocular sections, namely a nuclear association with cells of the corneal epithelium, trabecular meshwork, optic nerve and retina, the latter of which appeared to be restricted to inner retinal neurons. Confidence in the validity of these results is strengthened by the accompanying satisfactory Western blotting results, pre-adsorption testing and labelling patterns in putative positive control tissues.

## Conclusion

In conclusion, our results indicate that gene products of the 9p21 locus have defined and restricted patterns of expression in normal human and rodent ocular tissues. MTAP and p15^INK4B^ (CDKN2B) are relatively widely expressed, while p16^INK4A^ (CDKN2A) and p19^ARF^ are essentially undetectable with the tools we have used in healthy ocular tissues. The information provided in this study provide a basis for exploring how expression of these gene products may alter in glaucoma and other ocular diseases.

## Supporting Information

Figure S1 Evaluation of MTAP antibodies by Western immunoblotting. (A) Evaluation of three MTAP antibodies (Cell Signaling Technology, CST; Santa-Cruz, sc; ProteinTech, PT), by Western immunoblotting using GST-tagged, full length, recombinant, human MTAP protein (rMTAP). For each antibody tested, molecular weight markers were used to determine size of detected gel products (A; lane M). Lane 1, 100 ng rMTAP; Lane 2, 10 ng rMTAP; Lane 3, 1 ng rMTAP; Lane 4, 100 pg rMTAP. Single bands of the expected molecular weights (including GST-tag) are apparent for each of the antibodies. (B) Rat brain cortex (lane 1), liver (lane 2), optic nerve (lane 3) and retina (lane 4) samples probed for MTAP with the CST, sc and PT antibodies. Each antibody is able to detect a protein at the correct molecular mass for MTAP in the liver, optic nerve and retinal samples. Labelling for β-actin (housekeeping gene) and α-tubulin (neuronal tissue marker) are also shown.
(TIF)

Figure S2 Evaluation of MTAP antibodies in malignant pleural mesothelioma. In formalin-fixed, paraffin-embedded tissue sections, the MTAP antibody from Proteintech (PT Ab; A) robustly labelled inflammatory cells. In comparison, MTAP antibodies from Cell Signaling Technology (CST Ab; B) and Santa-Cruz (sc Ab; C) elicited weaker labelling of inflammatory cells (see arrows). Scale bar = 30 µm.
(TIF)

Figure S3 Evaluation of p16^INK4A^ antibodies, and p16^INK4A^ expression in rat tissues, by Western immunoblotting. (A) Evaluation of four p16^INK4A^ antibodies, from ProteinTech (PT), Sigma, Santa-Cruz (sc) and Abnova, by Western blotting, using GST-tagged, full length, recombinant, human p16^INK4A^ protein (rp16^INK4A^). For each antibody tested, molecular weight markers were used to determine size of detected gel products (A; lane M). Lane 1, 100 ng of rp16^INK4A^; Lane 2, 10 ng rp16^INK4A^. Single bands of the expected molecular weights (including GST-tag) are apparent for each of the tested antibodies except the Abnova one. (B) Rat brain cortex (lane 1), liver (lane 2), optic nerve (lane 3) and retina (lane 4) samples probed for p16^INK4A^ protein with the PT, Sigma, sc and Abnova antibodies. None of the antibodies detect proteins at the correct molecular mass for p16^INK4A^ in any rat tissue sample. Labelling for β-actin (house-keeping gene product) is also shown. (C) The Sigma and PT antibodies were further tested against retina (lanes 1–3) and optic nerve (lanes 4–6) samples obtained from three different rats. The presence of p16^INK4A^ protein is not detectable in any sample.
(TIF)

Figure S4 Evaluation of antibodies directed against p16^INK4A^ in cervical adenocarcinoma. In formalin-fixed, paraffin-embedded tissue sections, incubation with either the Santa-Cruz (sc; A), Proteintech (PT; B) or Sigma (C) antibodies identifies so-called ‘block’ or ‘diffuse’ immunolabelling of ductal tissue with a high signal-to-background, as compared to adjacent tissue (arrow). The staining intensity is greatest for the sc Ab and weakest for the Sigma antibody. The Abnova (D) antibody only inconsistently labels abnormal tissue and, additionally, produces nuclear labelling of adjacent tissue (red asterisk). Further examination of the Abnova antibody in cervical intraepithelial neoplasia reveals that the pattern of immunolabelling again differs from that obtained with the sc Ab (E) featuring weaker staining of the epithelium and robust nuclear localisation of numerous cells residing in the stromal tissue (F; red asterisk). Scale bar = 30 µm.
(TIF)

Figure S5 Representative images of p16^INK4A^ immunolabelling in human ocular tissues. In formalin-fixed, paraffin-embedded human eyes, no positive labelling for p16^INK4A^ is discernible in the corneal epithelium, trabecular meshwork (TM)/Schlemm’s canal (SC), or retina, irrespective of whether the Sigma (A–C) OR Proteintech (D–F) antibody is used. Scale bar: A, B, D, E, G, H = 15 µm; C, F, I = 30 µm. GCL, ganglion cell layer; INL, inner nuclear layer.
(TIF)

Figure S6 Representative images of p16^INK4A^ immunolabelling in rat retina and optic nerve. In formalin-fixed, paraffin-embedded rat retina (A–C) and optic nerve (D–F), no unambiguous, positive labelling for p16^INK4A^ is discernible either in retina or in the optic nerve using the Sigma (A, D) or Proteintech (B, E) antibodies. In contrast, the Abnova antibody robustly labels all cell nuclei within the eye, as highlighted in the presented images of retina (C) and optic nerve (F). Scale bar: A–D = 30 µm; E–H = 15 µm. GCL, ganglion cell layer; INL, inner nuclear layer; ONL, outer nuclear layer.
(TIF)

Figure S7 Representative images of p14^ARF^ immunolabelling using the antibody from CST. In formalin-fixed, paraffin-embedded sections of cervical intraepithelial neoplasia, p14^ARF^ expression is identified within nuclear inclusions (A). In formalin-fixed, paraffin-embedded human eyes, no positive nuclear labelling for p14^ARF^ is discernible in the corneal epithelium (B), trabecular meshwork (TM)/Schlemm’s canal (SC) (C), retina (D), unmyelinated optic nerve (E), or myelinated optic nerve (F). However, robust labelling of axons in the myelinated, but not unmyelinated, optic nerve is apparent. This labelling is likely non-specific. Scale bar = 15 µm.
(TIF)

Figure S8 Evaluation of P15^INK4B^ antibodies in the rat. (A) Evaluation of four P15^INK4B^ antibodies, from Cell Signaling Technology (CST), Neomarkers (Neo), Abcam and Santa-Cruz (sc), by Western blotting using GST-tagged, full length, recombinant human P15^INK4B^ (rP15^INK4B^) and P16^INK4A^ (rP16^INK4A^) proteins. M, molecular weight markers. Left-hand column, reactivity of antibodies to rP15^INK4B^ to determine specificity; right-hand column, reactivity of antibodies to rP16^INK4A^ in order to determine whether antisera bind non-specifically to related proteins. Left-hand column: lane 1, 100 ng of rP15^INK4B^; Lane 2, 10 ng rP15^INK4B^; Lane 3, 1 ng rP15^INK4B^ P; Lane 4, 100 pg rP15^INK4B^. Single bands of the expected molecular weights (for protein incorporating GST-tag) are apparent for each of the antibodies, except sc. Right-hand column: lane 1, 100 ng of rP16^INK4A^; Lane 2, 10 ng rP16^INK4A^; Lane 3, 1 ng rP16^INK4A^; Lane 4, 100 pg rP16^INK4A^. Abcam and, to a minor degree, Neo antibodies show non-specific reactivity with rP16^INK4A^. (B) Rat brain cortex (lane 1), liver (lane 2), optic nerve (lane 3) and retina (lane 4) samples probed for rP15^INK4B^ with CST, Neo, Abcam and sc antibodies. The CST antibody recognises a 15 kD protein in all tissue samples but the labeling for the other antibodies is disparate and variable. (C) Further testing of the sc antibody against different retina (lanes 1–3) and optic nerve (lanes 4–6) extracts reveals that the former antibody recognizes a distinct 35 kD protein in the retina with a labeling pattern which appears identical to that shown by the anti-rhodopsin antibody, Ret-P1. (D) Incubation of rat retina tissue sections with the sc antibody reveals immunoreactivity to be associated with photoreceptor outer segments (white arrow). This labelling pattern resembles that of RET-P1 labelling of rhodopsin (data not shown) and is unlikely to represent p15^INK4B^, which is typically localised to the nucleus of cells.
(TIF)

Figure S9 Evaluation of antibodies directed against p15^INK4B^ in cervical intraepithelial neoplasia and skin. In formalin-fixed, paraffin-embedded tissue sections, incubation with either the CST (A) or Santa-Cruz (B) p15^INK4B^ antibodies results in weak, diffuse immunolabelling of the cervical intraepithelial neoplastic lesion (red asterisk) and robust labelling of a subset of inflammatory cells in the underlying stroma (arrow). In contrast, incubation with the Abcam (C) p15^INK4B^ antibody yields a pattern of labelling that more closely resembles that of p16^INK4A^ (D), namely stronger staining of the CIN and negligible association with stromal components. In skin sections, the CST (E) and Santa-Cruz (F) antibodies labelled cutaneous squamous cells (arrows) Scale bar: A–D = 30 µm; E, F = 60 µm.
(TIF)
