# Supplementary figures and images for: Ocular Expression and Distribution of Products of the POAG-Associated Chromosome 9p21 Gene Region
Source: PLoS One. 2013 Sep 19;8(9):e75067. doi: 10.1371/journal.pone.0075067 (PMC3777912; doi:10.1371/journal.pone.0075067)

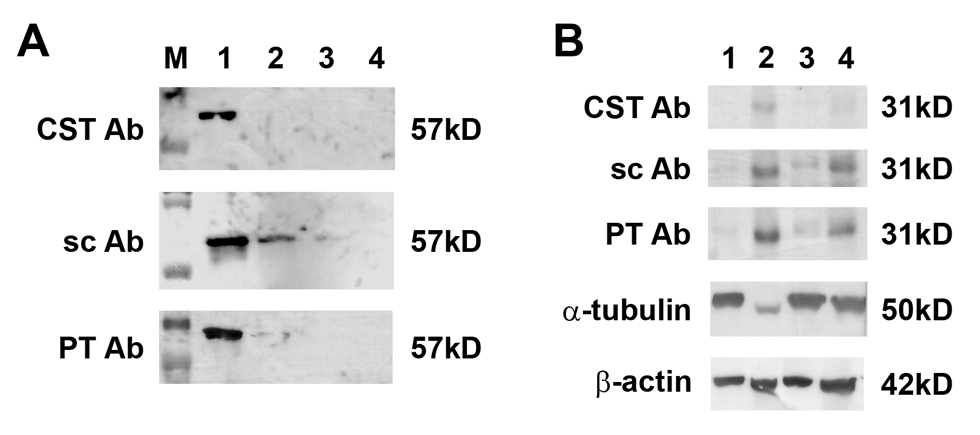

Supplement: Figure S1 — Evaluation of MTAP antibodies by Western immunoblotting. (A) Evaluation of three MTAP antibodies (Cell Signaling Technology, CST; Santa-Cruz, sc; ProteinTech, PT), by Western immunoblotting using GST-tagged, full length, recombinant, human MTAP protein (rMTAP). For each antibody tested, molecular weight markers were used to determine size of detected gel products (A; lane M). Lane 1, 100 ng rMTAP; Lane 2, 10 ng rMTAP; Lane 3, 1 ng rMTAP; Lane 4, 100 pg rMTAP. Single bands of the expected molecular weights (including GST-tag) are apparent for each of the antibodies. (B) Rat brain cortex (lane 1), liver (lane 2), optic nerve (lane 3) and retina (lane 4) samples probed for MTAP with the CST, sc and PT antibodies. Each antibody is able to detect a protein at the correct molecular mass for MTAP in the liver, optic nerve and retinal samples. Labelling for β-actin (housekeeping gene) and α-tubulin (neuronal tissue marker) are also shown. (TIF) [file pone.0075067.s001.tif]

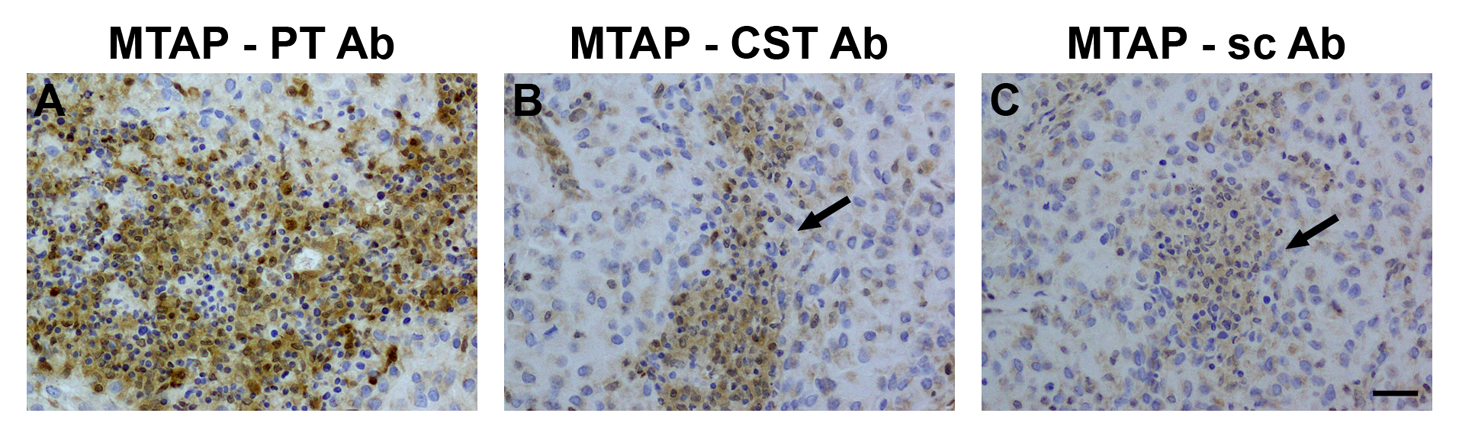

Supplement: Figure S2 — Evaluation of MTAP antibodies in malignant pleural mesothelioma. In formalin-fixed, paraffin-embedded tissue sections, the MTAP antibody from Proteintech (PT Ab; A) robustly labelled inflammatory cells. In comparison, MTAP antibodies from Cell Signaling Technology (CST Ab; B) and Santa-Cruz (sc Ab; C) elicited weaker labelling of inflammatory cells (see arrows). Scale bar = 30 µm. (TIF) [file pone.0075067.s002.tif]

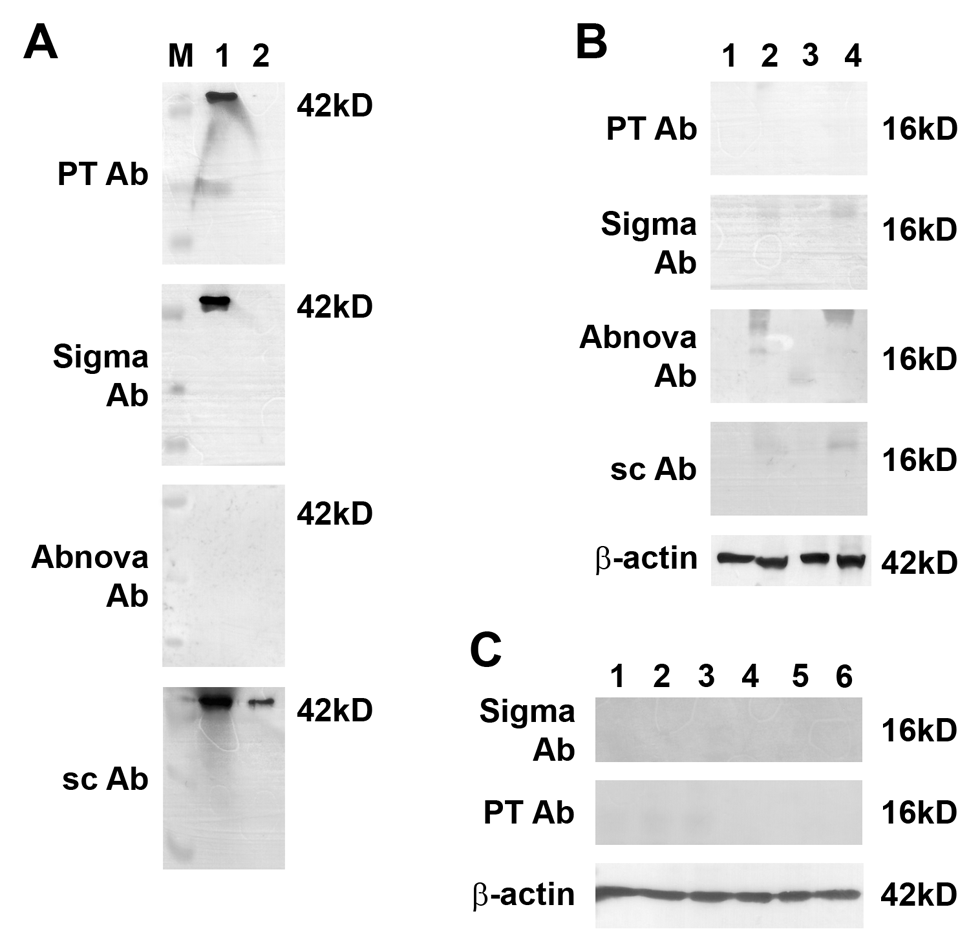

Supplement: Figure S3 — Evaluation of p16INK4A antibodies, and p16INK4A expression in rat tissues, by Western immunoblotting. (A) Evaluation of four p16INK4A antibodies, from ProteinTech (PT), Sigma, Santa-Cruz (sc) and Abnova, by Western blotting, using GST-tagged, full length, recombinant, human p16INK4A protein (rp16INK4A). For each antibody tested, molecular weight markers were used to determine size of detected gel products (A; lane M). Lane 1, 100 ng of rp16INK4A; Lane 2, 10 ng rp16INK4A. Single bands of the expected molecular weights (including GST-tag) are apparent for each of the tested antibodies except the Abnova one. (B) Rat brain cortex (lane 1), liver (lane 2), optic nerve (lane 3) and retina (lane 4) samples probed for p16INK4A protein with the PT, Sigma, sc and Abnova antibodies. None of the antibodies detect proteins at the correct molecular mass for p16INK4A in any rat tissue sample. Labelling for β-actin (house-keeping gene product) is also shown. (C) The Sigma and PT antibodies were further tested against retina (lanes 1–3) and optic nerve (lanes 4–6) samples obtained from three different rats. The presence of p16INK4A protein is not detectable in any sample. (TIF) [file pone.0075067.s003.tif]

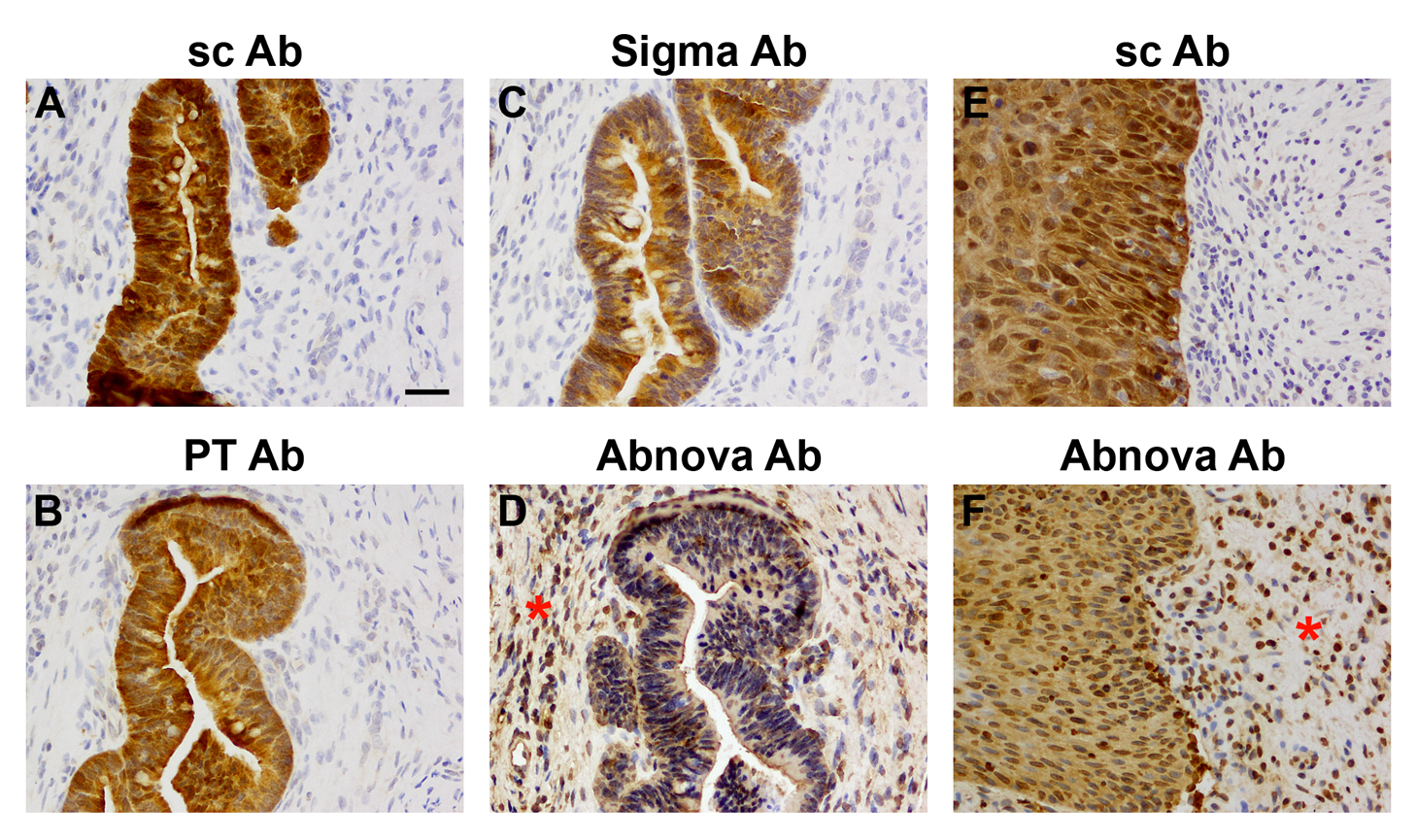

Supplement: Figure S4 — Evaluation of antibodies directed against p16INK4A in cervical adenocarcinoma. In formalin-fixed, paraffin-embedded tissue sections, incubation with either the Santa-Cruz (sc; A), Proteintech (PT; B) or Sigma (C) antibodies identifies so-called ‘block’ or ‘diffuse’ immunolabelling of ductal tissue with a high signal-to-background, as compared to adjacent tissue (arrow). The staining intensity is greatest for the sc Ab and weakest for the Sigma antibody. The Abnova (D) antibody only inconsistently labels abnormal tissue and, additionally, produces nuclear labelling of adjacent tissue (red asterisk). Further examination of the Abnova antibody in cervical intraepithelial neoplasia reveals that the pattern of immunolabelling again differs from that obtained with the sc Ab (E) featuring weaker staining of the epithelium and robust nuclear localisation of numerous cells residing in the stromal tissue (F; red asterisk). Scale bar = 30 µm. (TIF) [file pone.0075067.s004.tif]

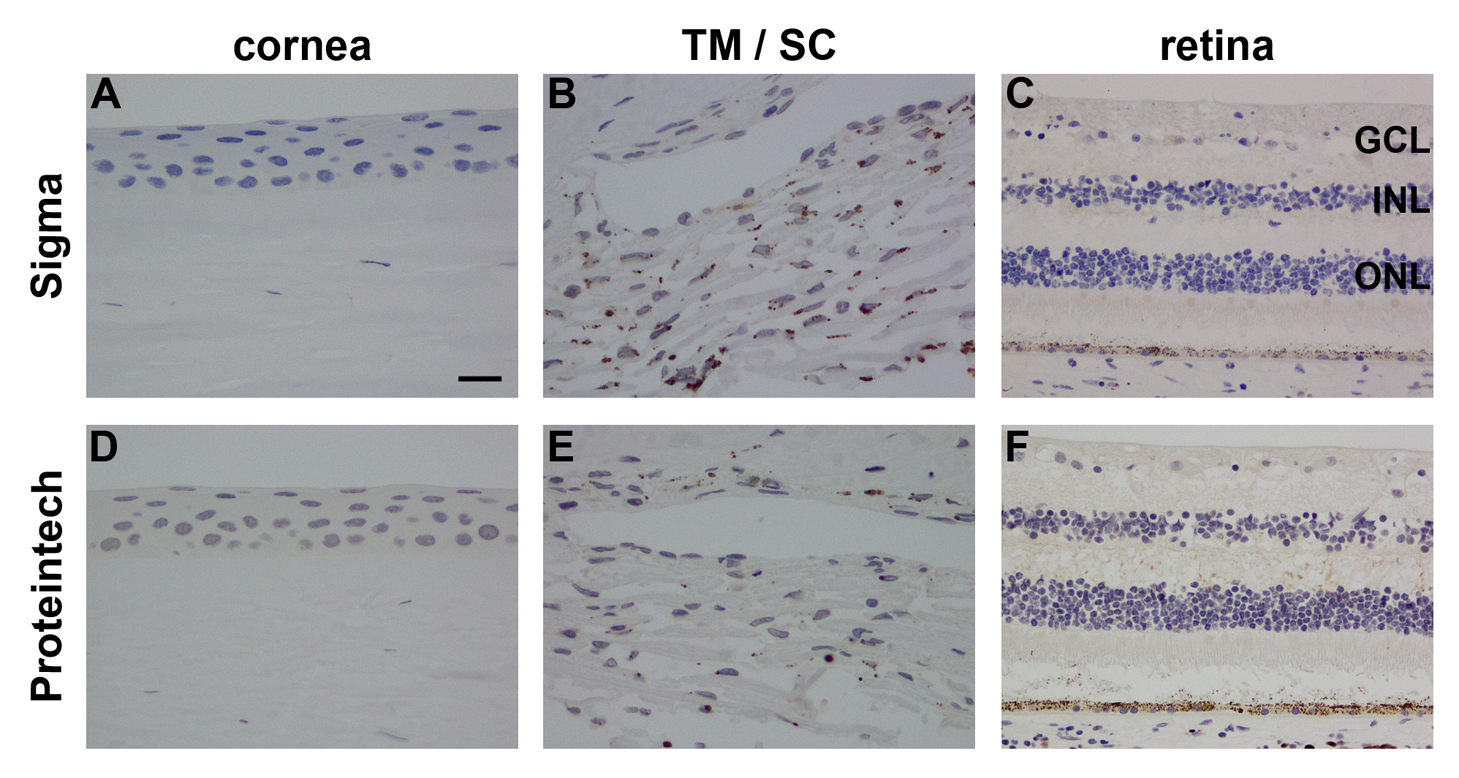

Supplement: Figure S5 — Representative images of p16INK4A immunolabelling in human ocular tissues. In formalin-fixed, paraffin-embedded human eyes, no positive labelling for p16INK4A is discernible in the corneal epithelium, trabecular meshwork (TM)/Schlemm’s canal (SC), or retina, irrespective of whether the Sigma (A–C) OR Proteintech (D–F) antibody is used. Scale bar: A, B, D, E, G, H = 15 µm; C, F, I = 30 µm. GCL, ganglion cell layer; INL, inner nuclear layer. (TIF) [file pone.0075067.s005.tif]

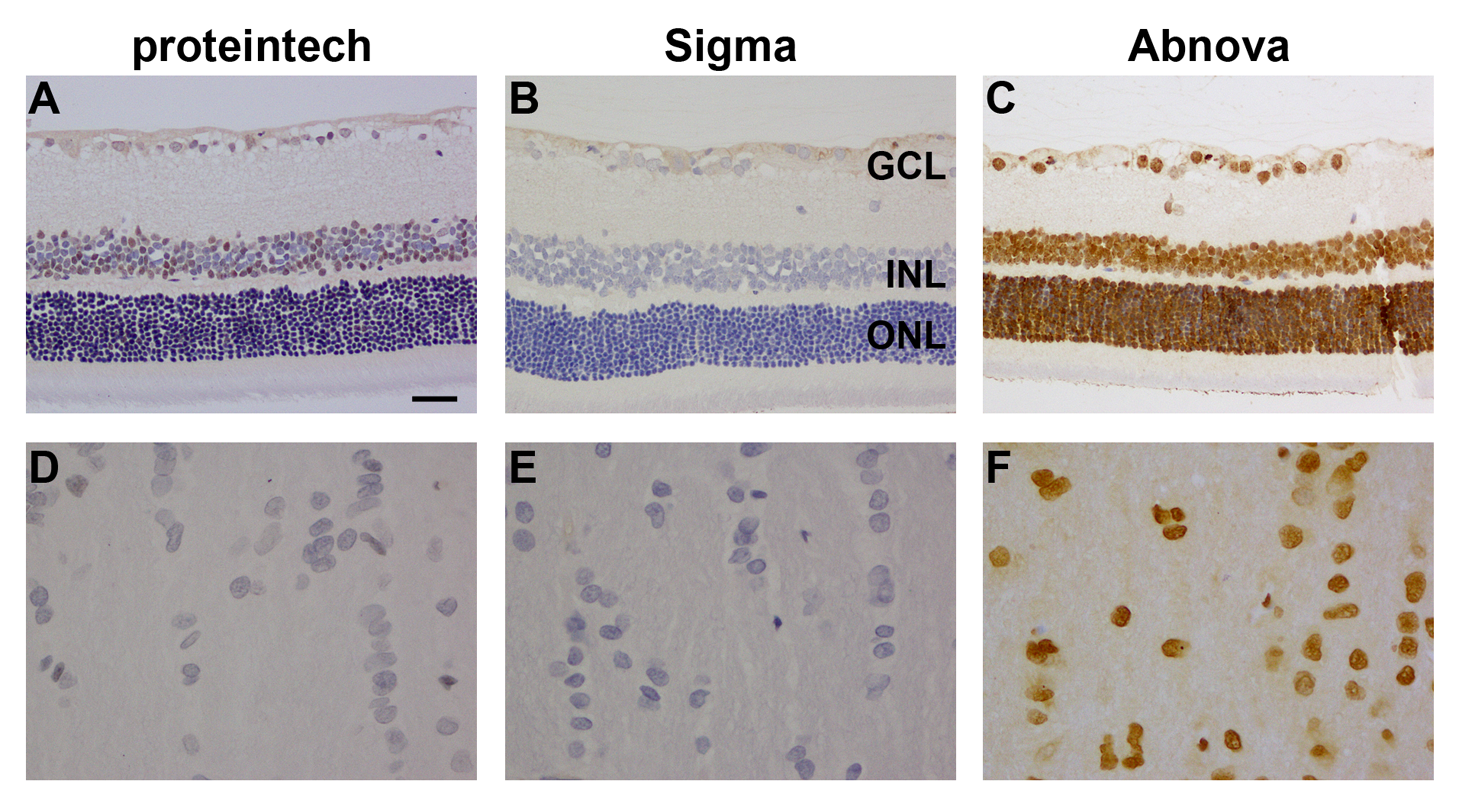

Supplement: Figure S6 — Representative images of p16INK4A immunolabelling in rat retina and optic nerve. In formalin-fixed, paraffin-embedded rat retina (A–C) and optic nerve (D–F), no unambiguous, positive labelling for p16INK4A is discernible either in retina or in the optic nerve using the Sigma (A, D) or Proteintech (B, E) antibodies. In contrast, the Abnova antibody robustly labels all cell nuclei within the eye, as highlighted in the presented images of retina (C) and optic nerve (F). Scale bar: A–D = 30 µm; E–H = 15 µm. GCL, ganglion cell layer; INL, inner nuclear layer; ONL, outer nuclear layer. (TIF) [file pone.0075067.s006.tif]

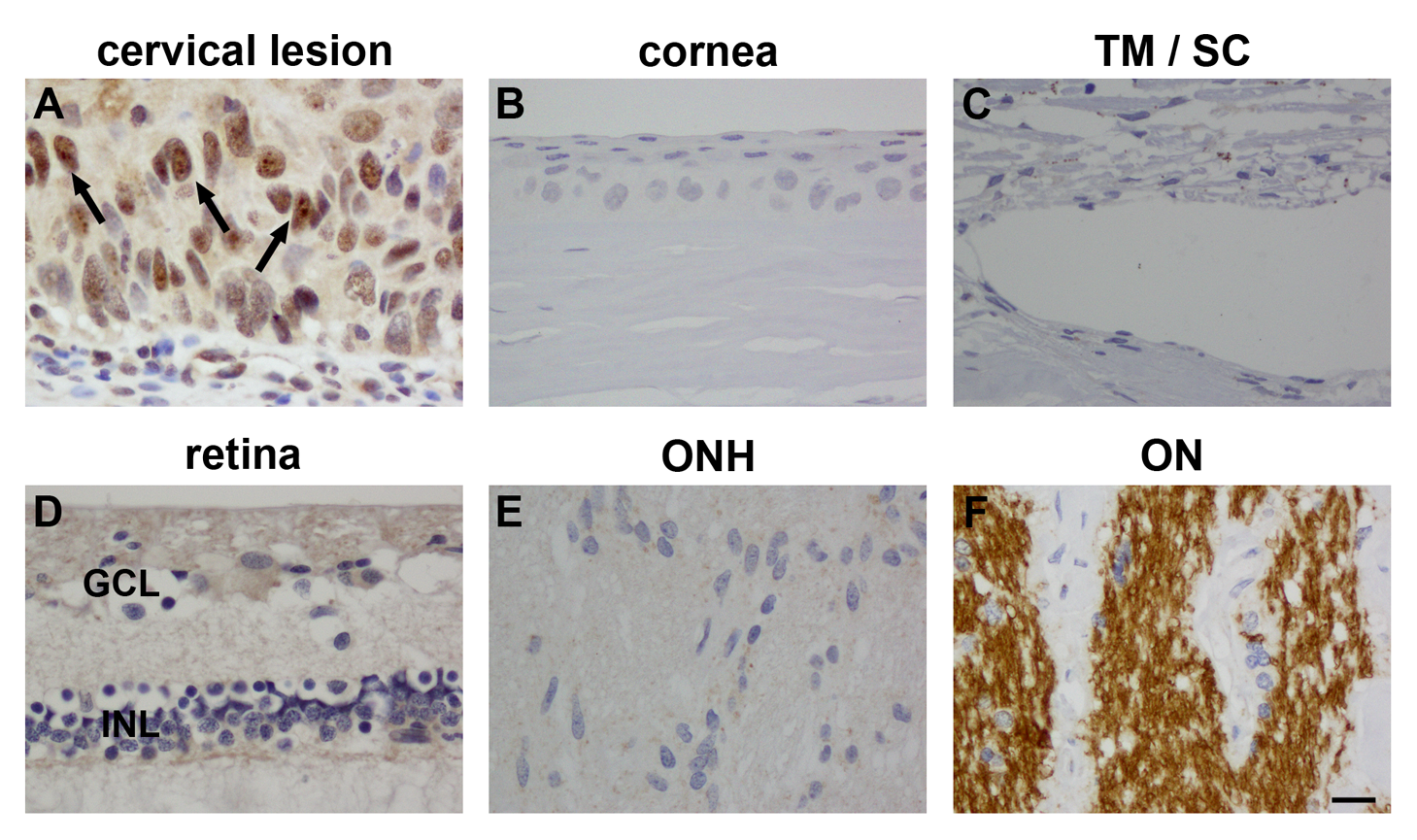

Supplement: Figure S7 — Representative images of p14ARF immunolabelling using the antibody from CST. In formalin-fixed, paraffin-embedded sections of cervical intraepithelial neoplasia, p14ARF expression is identified within nuclear inclusions (A). In formalin-fixed, paraffin-embedded human eyes, no positive nuclear labelling for p14ARF is discernible in the corneal epithelium (B), trabecular meshwork (TM)/Schlemm’s canal (SC) (C), retina (D), unmyelinated optic nerve (E), or myelinated optic nerve (F). However, robust labelling of axons in the myelinated, but not unmyelinated, optic nerve is apparent. This labelling is likely non-specific. Scale bar = 15 µm. (TIF) [file pone.0075067.s007.tif]

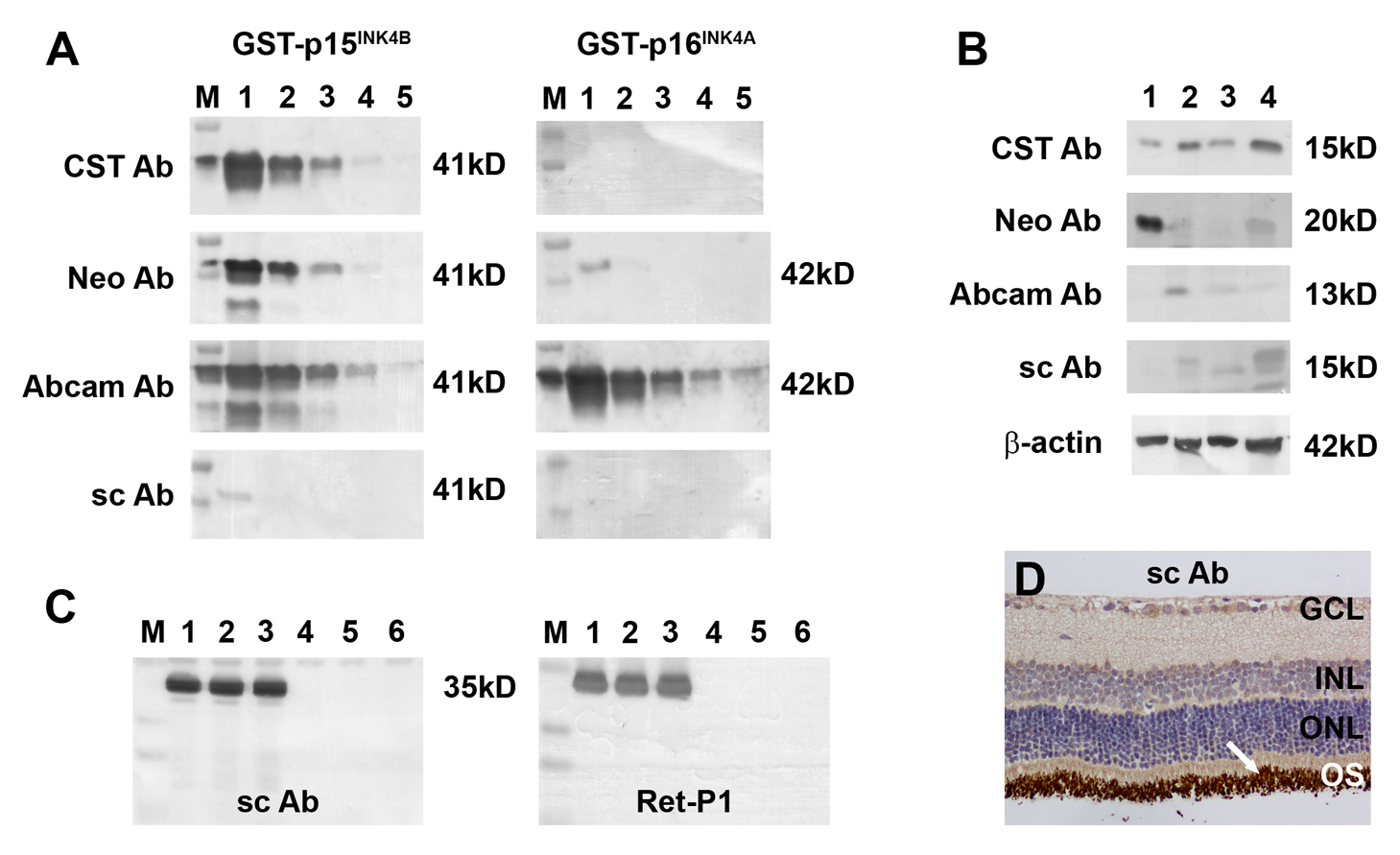

Supplement: Figure S8 — Evaluation of P15INK4B antibodies in the rat. (A) Evaluation of four P15INK4B antibodies, from Cell Signaling Technology (CST), Neomarkers (Neo), Abcam and Santa-Cruz (sc), by Western blotting using GST-tagged, full length, recombinant human P15INK4B (rP15INK4B) and P16INK4A (rP16INK4A) proteins. M, molecular weight markers. Left-hand column, reactivity of antibodies to rP15INK4B to determine specificity; right-hand column, reactivity of antibodies to rP16INK4A in order to determine whether antisera bind non-specifically to related proteins. Left-hand column: lane 1, 100 ng of rP15INK4B; Lane 2, 10 ng rP15INK4B; Lane 3, 1 ng rP15INK4B P; Lane 4, 100 pg rP15INK4B. Single bands of the expected molecular weights (for protein incorporating GST-tag) are apparent for each of the antibodies, except sc. Right-hand column: lane 1, 100 ng of rP16INK4A; Lane 2, 10 ng rP16INK4A; Lane 3, 1 ng rP16INK4A; Lane 4, 100 pg rP16INK4A. Abcam and, to a minor degree, Neo antibodies show non-specific reactivity with rP16INK4A. (B) Rat brain cortex (lane 1), liver (lane 2), optic nerve (lane 3) and retina (lane 4) samples probed for rP15INK4B with CST, Neo, Abcam and sc antibodies. The CST antibody recognises a 15 kD protein in all tissue samples but the labeling for the other antibodies is disparate and variable. (C) Further testing of the sc antibody against different retina (lanes 1–3) and optic nerve (lanes 4–6) extracts reveals that the former antibody recognizes a distinct 35 kD protein in the retina with a labeling pattern which appears identical to that shown by the anti-rhodopsin antibody, Ret-P1. (D) Incubation of rat retina tissue sections with the sc antibody reveals immunoreactivity to be associated with photoreceptor outer segments (white arrow). This labelling pattern resembles that of RET-P1 labelling of rhodopsin (data not shown) and is unlikely to represent p15INK4B, which is typically localised to the nucleus of cells. (TIF) [file pone.0075067.s008.tif]

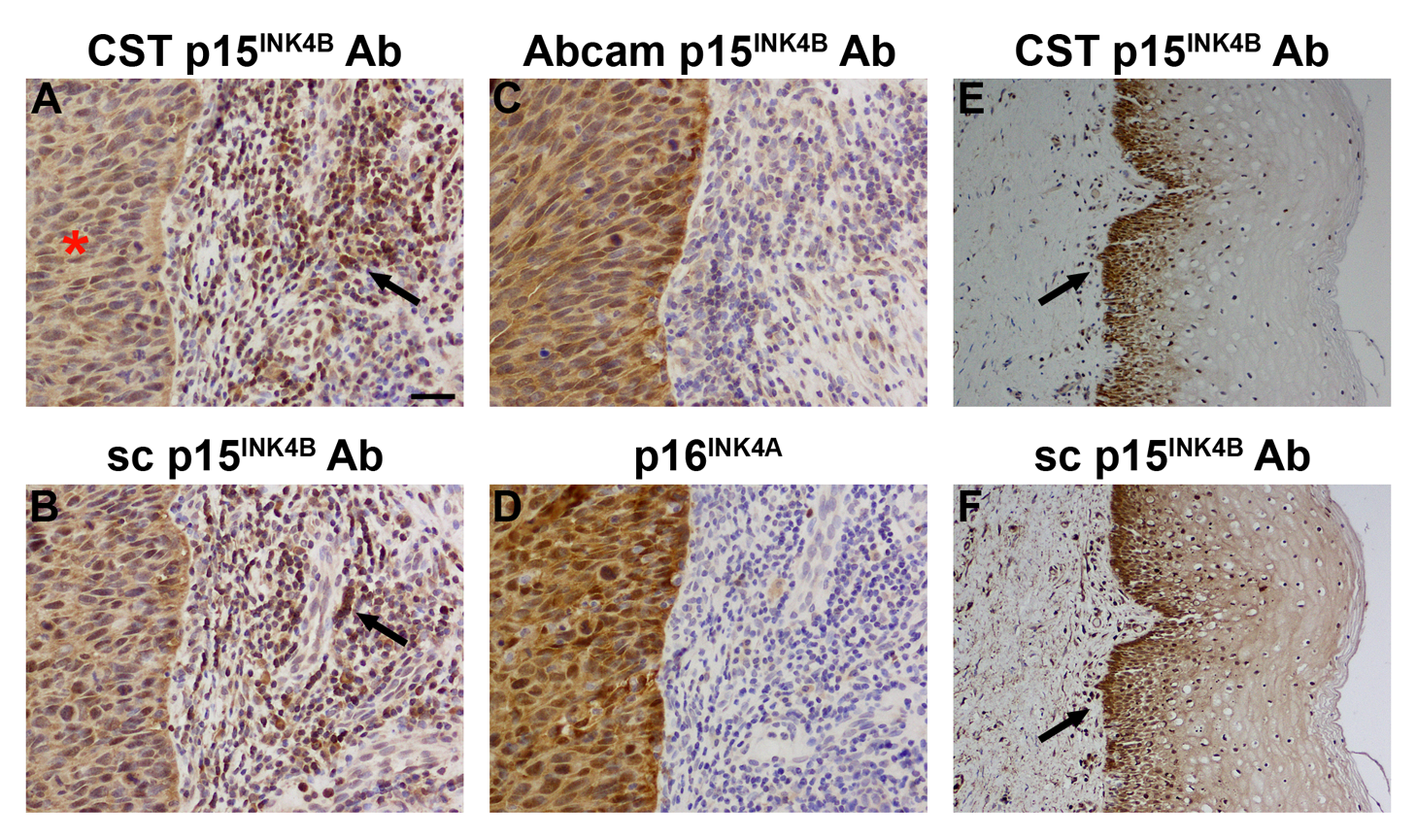

Supplement: Figure S9 — Evaluation of antibodies directed against p15INK4B in cervical intraepithelial neoplasia and skin. In formalin-fixed, paraffin-embedded tissue sections, incubation with either the CST (A) or Santa-Cruz (B) p15INK4B antibodies results in weak, diffuse immunolabelling of the cervical intraepithelial neoplastic lesion (red asterisk) and robust labelling of a subset of inflammatory cells in the underlying stroma (arrow). In contrast, incubation with the Abcam (C) p15INK4B antibody yields a pattern of labelling that more closely resembles that of p16INK4A (D), namely stronger staining of the CIN and negligible association with stromal components. In skin sections, the CST (E) and Santa-Cruz (F) antibodies labelled cutaneous squamous cells (arrows) Scale bar: A–D = 30 µm; E, F = 60 µm. (TIF) [file pone.0075067.s009.tif]
